# Molecular genetics of nicotine dependence and abstinence: whole genome association using 520,000 SNPs

**DOI:** 10.1186/1471-2156-8-10

**Published:** 2007-04-03

**Authors:** George R Uhl, Qing-Rong Liu, Tomas Drgon, Catherine Johnson, Donna Walther, Jed E Rose

**Affiliations:** 1 Molecular Neurobiology Branch, NIH-IRP, NIDA, Suite 3510, 333 Cassell Drive Baltimore, Maryland 21224, USA; 2 Dept of Psychiatry and Center for Nicotine and Smoking Cessation Research, Duke University, Durham NC 27708, USA

## Abstract

**Background:**

Classical genetic studies indicate that nicotine dependence is a substantially heritable complex disorder. Genetic vulnerabilities to nicotine dependence largely overlap with genetic vulnerabilities to dependence on other addictive substances. Successful abstinence from nicotine displays substantial heritable components as well. Some of the heritability for the ability to quit smoking appears to overlap with the genetics of nicotine dependence and some does not. We now report genome wide association studies of nicotine dependent individuals who were successful in abstaining from cigarette smoking, nicotine dependent individuals who were not successful in abstaining and ethnically-matched control subjects free from substantial lifetime use of any addictive substance.

**Results:**

These data, and their comparison with data that we have previously obtained from comparisons of four other substance dependent *vs *control samples support two main ideas: 1) Single nucleotide polymorphisms (SNPs) whose allele frequencies distinguish nicotine-dependent from control individuals identify a set of genes that overlaps significantly with the set of genes that contain markers whose allelic frequencies distinguish the four other substance dependent *vs *control groups (p < 0.018). 2) SNPs whose allelic frequencies distinguish successful *vs *unsuccessful abstainers cluster in small genomic regions in ways that are highly unlikely to be due to chance (*Monte Carlo *p < 0.00001).

**Conclusion:**

These clustered SNPs nominate candidate genes for successful abstinence from smoking that are implicated in interesting functions: cell adhesion, enzymes, transcriptional regulators, neurotransmitters and receptors and regulation of DNA, RNA and proteins. As these observations are replicated, they will provide an increasingly-strong basis for understanding mechanisms of successful abstinence, for identifying individuals more or less likely to succeed in smoking cessation efforts and for tailoring therapies so that genotypes can help match smokers with the treatments that are most likely to benefit them.

## Background

Extensive data from classical genetic studies indicate that vulnerabilities to substance dependence are complex traits with strong genetic influences. These genetic influences are largely shared by abusers of different legal and illegal addictive substances [1-4]. Nicotine dependence can be defined by Fagerstrom Test for Nicotine Dependence [5] or by DSM (diagnostic and statistical manual) criteria. Defined in either fashion, twin studies of nicotine dependence support strong heritability in the range of 40 – 70% [6-9].

Phenotypes relating to successful abstinence from smoking have also been studied in twin samples [10,11]. Success at achieving smoking abstinence displays heritability in the range of 40 – 60%. Some of this heritable component appears to overlap with heritable features of nicotine dependence and some does not.

Little current data indicates which specific genes contain variants that are likely to contribute to vulnerability to nicotine dependence and/or to success in abstaining from nicotine in formerly-dependent individuals. Linkage based genome scans for nicotine dependence, age of smoking onset and other nicotine dependence related phenotypes (*see discussion*) have identified a number of linkage peaks [12-20]. The most prominent linkage peaks from these efforts largely differ from sample to sample, however.

Identifying genomic markers for the allelic variants that contribute to nicotine dependence vulnerability should improve understanding of human addictions and aid efforts to match vulnerable individuals with the prevention and treatment strategies most likely to work for them. Adding information about genomic variations that help to distinguish successful quitters from non-successful quitters could have a significant impact on strategies to reduce the health burdens that cigarette smoking imposes.

To provide a basis for molecular genetic studies, we hypothesize that nicotine dependent participants in smoking cessation studies will display allele frequencies different from those identified in ethnically-matched control research volunteers. We postulate that we will find the most reliable results when the allele frequency differences between dependent and control individuals identify genes that we have previously identified in four prior genome wide association studies that compare allele frequencies in control individuals to those in individuals who are dependent on other abused substances. In addition, we hypothesize that successful cigarette quitters will display allele frequencies that differ from those found in ethnically-matched individuals who were unsuccessful at quitting. We thus address two research questions: 1) smokers *vs *non-smokers, with special interest in genes that overlap with genes identified in studies of dependence on other substances and 2) successful *vs *unsuccessful quitters.

**Figure 1:**
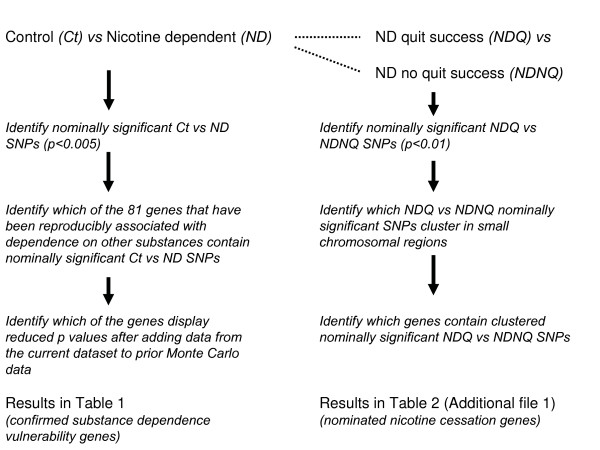
**Diagram outlining the analyses undertaken in this report**. (*left*) Comparisons between allele frequency assessments at 520,000 genomic SNPs in the whole group of European American nicotine dependent subjects who volunteered for inclusion in nicotine cessation trials in comparison to SNP frequency assessments for European-American control research volunteers without histories of any substantial use of any addictive substance. The preplanned analysis of this data focused on the extent to which these nominally positive SNPs added to the significance of the results of previously assembled convergent data from studies of other four other addict *vs *control comparisons. Genes for which the Monte Carlo significance increases (*eg *lower p values) after adding the current data to previously-obtained data are listed in Table 1. (*right*) Comparisons between allele frequency assessments at 520,000 genomic SNPs in two subgroups of the European American nicotine dependent research participants who volunteered for inclusion in nicotine cessation trials, described previously. NDQ subjects successfully abstained from smoking for at least 6 weeks after completion of therapeutic trials using nicotine and/or mecamylamine, NDNQ subjects did not abstain for this period. The preplanned analysis of this data focused on the extent to which the nominally-positive SNPs from this comparison clustered together in genomic regions that encoded genes in comparison to chance levels, assuming independence of SNP allelic frequencies. Genes that contain at least three nominally positive SNPs and are thus nominees to contain variants that participate in the genetic underpinnings of individual differences in smoking quit success are listed in [see additional file 1].

We thus now report 520,000 SNP genome wide association in pools of DNAs prepared from nicotine dependent European-American smoking cessation trial participants and control individuals. We compare genotypes from the entire group of nicotine dependent research participants to genotypes from European-American research volunteers free from any substantial lifetime use of any addictive substance. We also compare groups that displayed successful abstinence *vs *those who failed to display abstinence (Fig 1.).

## Results

SNP allele frequency assessments display modest variability. Standard errors for the variation among the four replicate studies of each DNA pool were +/- 0.035. Standard error for the variation among the pools studied for each phenotype group was +/- 0.028. Previous validating studies for these arrays have also revealed good fits between individual and pooled genotyping, with 0.95 correlations between pooled and individually-determined genotype frequencies [21-31]. The observed pool-to-pool standard deviations from these datasets thus indicate 0.94 and 1.0 power to detect 5 and 10% allele frequency differences with α = 0.05 in nicotine dependent *vs *control comparisons. We have 0.45 and 0.95 power to detect 5 and 10% allele frequency differences in successful *vs *unsuccessful quitters. Additional false negative results are likely to derive from the additional stringent requirement that four other samples each provide supporting evidence for the nicotine dependent *vs *control comparisons noted here.

We first focused on the first of the two research questions: 1) *smokers vs nonsmokers, with a special interest in the genes that have overlap with dependence on other substances*. When we compare allele frequencies in 134 nicotine-dependent *vs *320 control individuals, 88,937 of the 520,000 tested SNPs displayed t values that provide nominally-significant abuser *vs *control allele frequency differences at p < 0.005. These nominally-positive SNPs are positioned near clustered-positive SNPs from four other abuser-control comparisons to extents that are greater than expected by chance (Table 1). 4701 of these nominally-significant SNPs lie within 100 Kb of a cluster of nominally-positive SNPs from replicate African-American and European-American NIDA polysubstance abuser *vs *control comparisons. Monte Carlo p values for this convergence were 0.0002. Thus, only 2 of 10,000 Monte Carlo simulation trials that each began by selecting 88,937 random SNPs displayed so many nominally-significant results near the clustered positive results from the two NIDA samples. 2133 of the nominally-significant SNPs from the current nicotine dependent *vs *control comparison meet several criteria. They 1) lie near clusters of positive SNPs from both NIDA samples, 2) lie within annotated genes, 3) lie within genes that also supported by nominally-positive results from JGIDA methamphetamine abuser *vs *control comparisons and 4) lie within genes that are also supported by nominally-positive results from COGA alcohol dependent *vs *control comparisons. The Monte Carlo p value for the observed degree of convergence between the current and prior data is 0.018.

**Table 1:** Nicotine dependent *vs *control comparisons

***Gene/Cluster***	***Class***	***Chr***	***Bp***	***Rep Pos Snps***	***monte carlo p***	***Description***
CNTN6	CAM	3	1,280,415	11	0.00059	contactin 6
LRRN1	CAM	3	3,769,591	20	0.00007	leucine rich repeat neuronal 1
SEMA3C	CAM	7	80,111,952	9	0.00120	sema domain, immunoglobulin domain (Ig), short basic domain, secreted, (semaphorin) 3C
CSMD1a	CAM	8	3,184,850	11	0.00083	CUB and Sushi multiple domains 1
CSMD1b	CAM	8	3,653,990	13	0.00095	CUB and Sushi multiple domains 1
PTPRD	CAM	9	8,310,837	13	0.00047	protein tyrosine phosphatase, receptor type, D
LRRN6C$	CAM	9	29,153,017	6	0.00118	leucine rich repeat neuronal 6C
CDH13	CAM	16	81,647,004	11	0.00198	cadherin 13, H-cadherin (heart)
						
SIPA1L2	ENZ	1	228,796,685	16	0.00040	signal-induced proliferation-associated 1 like 2
PDE4D	ENZ	5	58,461,253	4	0.00329	phosphodiesterase 4D, cAMP-specific (phosphodiesterase E3 dunce homolog, Drosophila)
PDE1C	ENZ	7	31,648,914	8	0.00204	phosphodiesterase 1C, calmodulin-dependent 70 kDa
PRKG1a	ENZ	10	52,485,930	5	0.00299	protein kinase, cGMP-dependent, type I
PRKG1b	ENZ	10	52,986,999	10	0.00214	protein kinase, cGMP-dependent, type I
						
ELMO1	PROT	7	36,840,767	9	0.00243	engulfment and cell motility 1 (ced-12 homolog, C. elegans)
MICALCL	PROT	11	12,241,526	7	0.00115	MICAL C-terminal like
IMPACT*	PROT	18	20,182,039	9	0.0007	hypothetical protein IMPACT
						
GRM7	REC	3	6,934,982	5	0.00289	glutamate receptor, metabotropic 7
GPR154*	REC	7	34,383,589	4	0.00123	G protein-coupled receptor 154
HRH4*	REC	18	20,280,986	9	0.0007	histamine receptor H4
						
NFIB	TF	9	14,190,005	6	0.00274	nuclear factor I/B
						
KCNQ3*	CHA	8	133,172,472	5	0.00114	potassium voltage-gated channel, KQT-like subfamily, member 3
				4		
SLC9A9	TRANSP	3	144,947,291	12	0.00333	solute carrier family 9 (sodium/hydrogen exchanger), isoform 9
XKR5*	TRANSP	8	6,650,733	4	0.00063	XK, Kell blood group complex subunit-related family, member 5
ABCC4	TRANSP	13	94,600,083	5	0.0035	ATP-binding cassette, sub-family C (CFTR/MRP), member 4
						
PTHB1	DIS	7	33,369,755	21	0.00250	parathyroid hormone-responsive B1
						
ACTN2	STR	1	233,147,888	5	0.00016	actinin, alpha 2
OC90*	STR	8	133,172,472	5	0.00114	otoconin 90
						
HHLA1*	OTHER	8	133,172,472	12	0.00114	HERV-H LTR-associating 1
DEFB1*	OTHER	8	6,650,733	5	0.00063	defensin, beta 1
FGF14	OTHER	13	101,764,771	12	0.003	fibroblast growth factor 14
A2BP1	OTHER	16	6,603,645	9	0.00171	ataxin 2-binding protein 1
OSBPL1A	OTHER	18	20,182,039	11	0.0007	oxysterol binding protein-like 1A

Nicotine dependent *vs *control comparisons from the current work add support to previous addict *vs *control association observations in specific genes. Genes and classes of genes that contain nominally positive (p < 0.005) SNPs in comparisons between nicotine dependent(*n *= 139) and control (*n *= 320) individuals in the current study and enhance the significance of previously-obtained whole genome association results for addiction. To be included in this list, the data from the current comparison needs to improve the nominal significance of 100000 Monte Carlo simulation trials by > 10 trials when the current data is added to data from four prior samples. Four prior samples are comprised of genes previously nominated to play roles in addiction based on reproducible nominally positive allele frequency differences between European-American, African-American and Japanese individuals who are dependent on illegal substances or alcohol. Genes in this table this contain: 1) SNPs that display p < 0.005 significance for differences between nicotine dependent and control individuals in the present study 2) clustered SNPs that displayed significant (p < 0.05) differences between both European-American and African-American NIDA polysubstance abusers *vs *controls in previous studies 3) SNPs that displayed p < 0.05 significance in 100k association genome scans of alcohol dependent *vs *control individuals (COGA [55]) and 4) SNPs that displayed p < 0.05 significance in 100k association genome scans of methamphetamine dependent *vs *control individuals (JGIDA [56]).

Genes are identified when positive SNPs lie 1) within the gene's exons or introns or 2) in 3' or 5' flanking sequences that lay within 100 Kb of an annotated exon or extensions of the currently-annotated exons as described [22]. Genes are grouped by the class of the function to which they contribute: "CAM" cell adhesion, "ENZ" enzymes, "PROT" protein processing, "REC" receptors, "TF" transcriptional regulation, "CHA" channels, "TRANSP" transporters, "DIS" disease associated, "STR" structural, "OTHER" other functions. Chromosome number and initial chromosomal position for the cluster (bp, NCBI Mapviewer Build 35.1) are listed. Monte Carlo p values come from 100,000 simulation trials. In each trial, randomly selected sequences lying within randomly selected gene sequences of the same length displayed by the actual genomic segments analyzed here were assessed to determine whether or not they contained at least the number of positive SNPs actually identified for each gene cluster and gene. The frequency of trials in which at least the observed numbers of nominally-positive SNPs were identified in each of the four samples studied here was recorded to provide an empirical p value. Several genes are identified by the same clusters of positive SNPs; these genes are indicated with asterisk symbols. Several genes, identified in several lines of Table 1, contain multiple clusters of reproducibly positive SNPs; the clusters are designated by suffixes a, b etc. We note that the requirements for nominally-significant association signals in each of five samples and increasing significance based on data from the current nicotine dependent vs control comparisons are likely to increase the number of false-negative results; interesting genes that receive support from only four samples are not listed here, for example.

The results of the nicotine-dependence *vs *control comparisons from the current study provide substantial confirmation for a number of genes in several gene classes that have been nominated and confirmed in prior addict *vs *control studies. Seven previously nominated genes related to cell adhesion processes, CNTN6, LRRN1, SEMA3C, CSMD1, PTPRD, LRRN6C and CDH13 each receive additional support from 100,000 Monte Carlo simulation trials. The convergence between current and previously-obtained data suggest that allelic variants in these genes are thus likely to contribute to individual differences in vulnerability to a variety of addictive substances (Table 1). Four genes related to enzymatic activity, SIPA1L2, PDE1C, PDE4D and PRKG1 each receive similar support. Genes involved in protein processing, a transcriptional regulator, and genes involved in channel, transporter, structural, disease and other processes receive similar support. Three G-protein coupled receptors, the GRM7 metabotropic glutamate receptor, the orphan GPR154 and the HRH4 histamine receptor also receive such support. Each of these genes, taken individually, is thus supported by data from studies of individuals selected on the basis of their dependence on illegal substances (largely cannabis, stimulants and opiates), methamphetamine, alcohol and tobacco.

*Controls for occult stratification among these subjects and poor technical quality *in the nominally-positive SNPs identified here fail to provide alternative explanations for the positive results of comparisons between smokers and controls. Only 837 of the nominally-positive SNPs from the smoker-control comparisons display large allele frequency differences between European- and African-American control individuals. This number is smaller than the 2,223 SNPs that would be expected to have such properties if they were selected by chance. Only 158 of the nominally-positive SNPs from the smoker-control comparisons in these data lie among the SNPs that display the largest variation between pools in data from this and other studies using the same arrays. This number is also smaller than chance values. These comparisons thus fail to support the alternative hypotheses that either occult ethnic stratification in these samples or technical problems with assays for these SNPs provided the basis for the overall results reported here.

We next focused on the second research question: 2) *successful vs unsuccessful quitters*.

In comparing data from successful *vs *unsuccessful quitters, we identified 4,570 SNPs whose allele frequencies differ between these two groups with t values for these differences that yield nominal p values < 0.01. The nominally-positive SNPs from comparisons between successful *vs *unsuccessful quitters cluster together to extents much greater than expected by chance if their allelic frequencies were independent of each other (Monte Carlo p < 0.00001). 944 of the 4,570 nominally-positive SNPs lay in 224 clusters in which each positive SNP lay within 100 Kb of at least one other positive SNP. We would anticipate such clustering if many of these reproducibly-positive SNPs identified haplotypes that were present in different frequencies in our samples of successful *vs *unsuccessful quitters, but not if they represented chance independent observations. We defined clusters as chromosomal sites where 1) three or more reproducibly-positive SNPs were positioned within 0.1 Mb of each other and 2) reproducibly-positive SNPs assessed by two different array types were represented, so that all positive data did not come from just *Nsp *I or from *Sty *I arrays.

*The nominally-positive SNPs from successful vs unsuccessful quitter comparisons that cluster together on small chromosomal regions also cluster together in regions that are annotated as genes *to extents much greater than chance if they represented independent observations (Monte Carlo p < 0.00001 for both).

Neither *controls for occult stratification nor for poor technical quality *explain the nominally-positive SNPs from the successful *vs *unsuccessful quitter comparisons. The SNPs that display the largest allele frequency differences between European- and African-American controls and the SNPs that display the largest between-pool variances do not overlap with those that distinguish successful *vs *unsuccessful quitters at levels significantly larger than those anticipated by chance (131 *vs *114 and 143 *vs *114, respectively).

Haplotypes that were present at different frequencies in the successful *vs *unsuccessful quitters by chance, not based on ethnic stratification, could conceivably contribute to some of this clustering; we thus view the results reported here [see additional file 1] as nominally-positive genes. Nevertheless, the 221 genes identified by these clustered positive results represent a highly interesting set [see additional file 1]. Seventeen of these genes produce products related to cell adhesion, 39 genes' products relate to enzymatic activities, 37 encode receptors and/or G-protein mechanisms, 5 encode channels, 27 encode transcriptional regulators, 9 genes' products are involved in mechanisms for Mendelian disorders, 12 encode structural proteins, 4 encode proteins involved with vesicle function, 5 encode transporters, 32 encode genes involved with DNA, RNA or protein processing and 34 are genes about which so little is known that we cannot confidently place them in a functional class.

## Discussion

The molecular genetic observations reported here are consistent with substantial heritabilities for nicotine dependence *vs *nondependence and for successful abstinence *vs *unsuccessful abstinence, as suggested by classical genetic studies [3,6-8,10,11]. The current data support the idea that nicotine dependence shares substantial heritable features with dependence on other addictive substances. These molecular results also support the idea that some of the genetics of nicotine dependence overlaps with the genetic underpinnings of successful abstinence while some is independent.

Several *genes contain SNPs whose allelic frequencies distinguish nicotine dependent from control individuals*. We have focused on the 30 genes for which the differences between dependent and control individuals enhance the convergence of results previously obtained from four other abuser *vs *control whole genome association studies. The identification of allelic associations within so many genes that encode cell adhesion and extracellular matrix molecules support important roles for neuronal connectivities and memory-like functions in individual differences in vulnerabilities to addictions [32]. Data for each of these 30 genes provides new information about vulnerability to nicotine dependence. However, the approach that we use here does bias against genes that may contribute to vulnerability to nicotine alone. Failure to be included on this list should not be taken to exclude involvement in nicotine dependence of genes, such as those that encode nicotine metabolizing enzymes, that have been associated with nicotine dependence in previous studies [33].

Nominally-significant linkage of a number of genomic markers to smoking phenotypes has been identified. Five reports on data from the Framingham Heart Study (smoking rate) [16], (> 0 cigarettes/day) [34], (>0.0138 pack/years) [12], two reports on data from the Collaborative Study on the Genetics of Alcohol [17,35], (cigarettes/day for 1 year) [13], ("habitual smoking > 20 cigarettes/day for > 6 months) [36], two reports on data from a sample recruited in Christchurch, New Zealand (Fagerstrom) [18,19], two reports on data from a sample recruited in Richmond, Virginia (Fagerstrom) [18,19], as well as single reports on linkage data from Mission Indians (smoking daily > 1 mo; smoking > 10 cigarettes/day > 1 year) [14], Oregon Smoking in Families Study (Fagerstrom and nicotine dependence measures) [20], and Yale Anxiety Clinic pedigree members (> 20 cigarettes/day for >1 year or > 10 cigarettes/day for > 10 years) [15] add to the list of markers with nominally-significant linkage to smoking phenotypes. Support for cadherin 13 is enhanced by the linkages to D16S422 and D16S684 identified by Straub and by Sullivan [18] in New Zealand samples (*also, see below*).

The *genes that contain multiple clustered nominally-positive SNPs that distinguish successful quitters from those who could not abstain *successfully also represent an interesting group. This list of genes includes several that contain SNPs whose allelic frequencies also distinguish nicotine dependent from control individuals. Cadherin 13 is a cell adhesion molecule identified in both comparisons and in the linkage results noted above. Cadherin 13 is glycosyl-phosphatidylinositol (GPI) anchored and likely to be localized to lipid raft membrane domains where it produces homophilic interactions with other CDH 13 molecules and heterophilic interactions with ligands that include adiponectin hexamers and low density lipoproteins [37-40]. Ligand interactions with CDH13 activate signaling pathways including those that alter intracellular Ca2+ and tyrosine kinase, Erk 1/2 kinase, RhoA/ROCK and Rac pathways and NFkB [37-40]. Cadherin 13 can inhibit neurite extension from select neuron populations both as a substratum and as a soluble recombinant protein [41]. Expression is documented in neurons located in interesting human brain regions including frontal cortex, amygdala and ventral midbrain [42].

The cyclic G dependent protein kinase gene is identified in both comparisons. This gene is widely and multifocally expressed in brain in cells including neurons [43]. Proper PRKG1 expression is important for proper brain development [44]. Variants in this gene can lead to marked differences in behaviors of *drosophila *[45]. Nitric oxide can dramatically modulate brain cGMP systems, suggesting that these systems may provide some of the primary targets for the products of nitric oxide synthases (NOS). Mnemonic and addictive functions can each be altered by changes in cGMP-dependent protein kinase and/or NOS [46-48].

In addition to CDH13 and PRKG1, 214 additional genes are identified by the clustered positive results that we nominate from comparisons of treatment-seeking individuals who successfully *vs *unsuccessfully abstain from smoking. Sixteen of these additional genes produce products related to cell adhesion, 32 genes' products relate to enzymatic activities, 37 encode receptors and/or G-protein mechanisms, 27 encode transcriptional regulators and others encode channels, gene products involved in mechanisms for Mendelian disorders, structural proteins, proteins involved with vesicle function, transporters, genes involved with DNA, RNA or protein processing and genes of unknown functions. These genes, taken together, should be considered nominees to contain variants that could play roles in the genetic underpinnings of successful abstinence from smoking. We can confidently exclude the probability that technical features contribute to the genes identified by the quitter *vs *nonquitter comparisons. With the modest sample sizes reported here, however, we cannot exclude contributions from random differences in haplotype distributions between these two groups. Further studies will be necessary to confidently identify which of the individual genes nominated in this study display replicable results.

The *current observations contain significant limitations *that should be considered in their interpretation. First, the modest sizes of the samples used for these studies provide moderate power, at best, to detect gene variants related to nicotine dependence and successful quitting. As noted in the power calculations, the number of false negative results is likely to be higher for allelic variants that produce small effects. Second, in conjunction with the modest sample sizes, we have also imposed stringent requirements for the genes listed in Table 1. Each of these genes is required to contain SNPs that display nominally significant abuser/control allele frequency differences in four prior samples, and also to display enhanced Monte Carlo p values when the current dataset is added to previously-obtained datasets. While these analyses reduce the probability that these genes will represent false positives, it is also likely to lead to many false-negative results. If we even allow genes whose Monte Carlo probabilities are not reduced by adding the current data to be included, most of the genes previously supported by the four prior datasets for other addictions [22,31,49] would also be included in Table 1 (*data not shown*).

Third, the current data for nicotine-dependent *vs *control comparisons uses well-characterized research volunteer European-American control samples that overlap substantially with those used for comparisons with European-American polysubstance abusers. While we have no evidence for any substantial occult differences between the underlying European-American research participants sampled in North Carolina and those sampled in Maryland, differences that cannot be detected by our extensive genomic control procedures are not inconceivable. In addition, these results are thus not totally independent from those in the substance abuser *vs *control comparisons to which the current nicotine dependence *vs *control data are compared. Since the control group used here overlaps with only one of the control groups used for the previous datasets, we believe that this potentially confounding influence is unlikely to have a large impact on the overall results.

Fourth, as noted above, the list of genes that distinguish successful from unsuccessful quitters should be considered as a list of nominees, in light of the modest power available for this comparison and the likely inclusion of false-positive results on this list. In spite of this caution, however, we do find that this list of these genes overlaps with the genes that distinguish nicotine-dependent from control individuals. We also note that these positional cloning results identify genes whose products can substantially impact animal models for relapse. We identify corticotrophin releasing hormone (CRH), for example. Stressors of several sorts elevate CRH and lead to dramatically elevated relapse in animal models [50]. We also identify a gene cluster that contains two melanocortin G protein coupled receptors. We have never consistently identified CRH or melanocortin receptor genes in our studies comparing addicts to controls. These CRH and melanocortin receptor genes are thus candidates to contribute to the genetic influences on quitting success that may be independent of the genetic influences on nicotine dependence. Fifth, there are modest to moderate differences in the gender and age of nicotine-dependent *vs *control research volunteers studied here. While we have focused only on data from autosomal regions in these analyses and sought its replication in studies of several other addict *vs *control samples in ways that are likely to minimize these influences, they may not be able to eliminate them. Both nicotine-dependent and control groups are also sufficiently old to have passed through the vast majority of the ages of risk of development of nicotine dependence. Nevertheless, it is conceivable that the modest age differences in the samples studied here might have contributed modestly to some of the observed results. Sixth, in order to enhance the likelihood that the genes identified in the dependent *vs *control comparisons represent true positive observations, we have focused on gene variants that are also identified in other comparisons between individuals who are dependent on other substances *vs *controls. This strategy may reduce the novelty of the list of genes reported here, though these findings do provide novel information concerning the possible roles of variants in these genes in vulnerability to nicotine dependence as opposed to dependence on other substances. We can compare current data to very recent reports that identify SNPs whose allelic frequencies differ between dependent *vs *nondependent smokers [51,52]. Three hundred thirty-one and 623 of the SNPs that distinguish nicotine dependent *vs *control individuals and 16 and 25 of the SNPs that distinguish successful *vs *unsuccessful quitters lie within 10 and 100 kb of one of these candidate genes. These SNPs thus provide modest additional support to findings reported at the ADRBK2, AVPR1A, BDNF, CCK, CHRNA10, CHRNA2, CHRNA4, CHRNA5, CHRNA6, CHRNA7, CHRNB2, CHRNG, CLCA1, CLTCL1, CNR1, CTNNA3, DBH, DDC, DRD1, DRD3, FBXL17, FMO1, FMO4, FTO, GABBR2, GABRA4, GABRB2, HTR1A, HTR5A, KCNJ6, NPY, NRXN1, OPRD1, OPRK1, PDYN, PENK, PIP5K2A, POMC, SLC6A3, SLC6A4, TRPC7 and VPS13A loci [51,52].

## Conclusion

Repeated studies in carefully selected samples will be necessary to confirm many of these observations. Larger samples that study effects of single pharmacologic treatments may also identify genes whose influences are specific to particular treatments. The current data not only nominates candidate for replication in further samples, however. Taken as a whole, it provides molecular genetic support for the idea that ability to abstain from nicotine has polygenic genetic components that overlap, in part, with those that contribute to vulnerability to nicotine dependence. This work also supports overlaps between the polygenic molecular genetic determinants that predispose to nicotine vulnerability and those that predispose to addictions to other legal and illegal addictive substances. Each of these features thus provides support for further elucidation of genetic variants that are associated with smoking cessation success. Each of these results provides promise that we may be able to begin to use such data to help match treatments with those most likely to benefit from them in the relatively near future.

## Methods

### Experimental subjects

Study participants of self-reported European ancestry recruited in the Raleigh-Durham metropolitan area by advertising and word of mouth provided informed consents for studies of smoking cessation, averaged age 44 and were 45% female. These participants reported an average of 25 years of smoking, displayed initial Fagerstrom Test for Nicotine Dependence (FTND) [5] scores that averaged 6.4 and provided screening carbon monoxide levels that averaged 34.7. Participants received oral mecamylamine (10 mg/day) and either active (21 mg/24 h) or placebo nicotine skin patches for two weeks before the target quit-smoking date. After the quit-date, participants were randomly assigned to groups that received mecamylamine (10 mg/day) *vs *matching placebo and 21 mg/24 h *vs *42 mg/24 h nicotine skin patch doses to test how mecamylamine might improve effectiveness of nicotine replacement therapy. Behavioral support and self-help quitting manuals were also provided. Fifty-five study participants reported continuous abstinence from smoking when assessed 6 weeks after the quit date. 79 participants were not abstinent at the 6 week time point. Data from these individuals was compared to data from 320 control study participants of self-reported European-American ancestry recruited in Baltimore by advertising and word of mouth who also provided informed consents, averaged age 31, were 36% female and reported no substantial lifetime histories of use of any addictive substance [21,53,54].

### DNA preparation, pooling and analysis

Genomic DNA was prepared from blood [21,53,54], carefully quantitated and combined into pools representing 13 – 20 individuals of the same ethnicity and phenotype. Hybridization probes were prepared from the genomic DNA pools as described (Affymetrix Genechip Mapping Assay Manual) with precautions to avoid contamination that included use of dedicated preparation rooms and hoods. 50 ng of each pooled genomic DNA was digested by *Sty*I or by *Nsp*I, ligated to appropriate adaptors and amplified using a GeneAmp PCR System 9700 (Applied Biosystems, Foster City, CA) with a 3 min 94°C hot start, 30 cycles of 30 sec 94°C, 45 sec 60°C, 15 sec at 68°C and a final 7 min 68°C extension. PCR products were purified (MinElute™ 96 UF kits, Qiagen, Valencia, CA). PCR products were quantitated and 40 μg were digested for 35 min at 37°C with 0.04 unit/μl DNase I. The 30–100 bp fragments resulting from DNAse treatments were end-labeled using terminal deoxynucleotidyl transferase and biotinylated dideoxynucleotides and hybridized to the appropriate *Sty *I or *Nsp *I early access Mendel^® ^microarrays (Affymetrix, Santa Clara, CA). Arrays were stained, washed and scanned as described (Affymetrix Genechip Mapping Assay Manual) using immunopure strepavidin (Pierce, Milwaukee, WI), biotinylated antistreptavidin antibody (Vector Labs, Burlingame, CA) and R-phycoerythrin strepavidin (Molecular Probes, Eugene, OR). Fluorescence intensities were quantitated using an Affymetrix array scanner as described [21].

### Identification of positive SNPs

Allele frequencies for each SNP in each DNA pool were assessed based on hybridization to the 12 "perfect match" cells on each of four arrays from replicate experiments, as described [31,55]. In brief, each cell's value was analyzed by subtracting background fluorescence intensities and normalizing background-subtracted values to the values for the highest intensities on each array. We averaged the data from the 12 perfect match cells for A and B alleles for each SNP. To facilitate comparison of data from multiple arrays, we derived the arctangent of the ratio between hybridization intensities for A and B alleles for each array. We then averaged these arctan A/B values for the four replicate arrays that assessed genotype frequencies for each pool. We calculated the mean arctan A/B ratios for nicotine dependent *vs *control individuals (and for quitters *vs *nonquitters). We divided the mean arctan A/B ratio for abusers (or quitters) by the mean arctan A/B ratio for controls (or nonquitters) to form abuser/control (or quitter/nonquitter) ratios. We generated a "t" statistic for the differences between abusers and controls or quitters and nonquitters using the formula described previously [22,31,55]. "Nominally significant" SNPs display t values with p < 0.005 for nicotine dependent *vs *control comparisons and p < 0.01 for quitter *vs *nonquitter comparisons, respectively. We thus set a relatively strict preplanned criterion for the first comparison that confirms genes with good confidence. We set a more modest criterion, with lower levels of confidence, for the second comparison that nominates genes that merit replication studies. We deleted data from SNPs on sex chromosomes and SNPs whose chromosomal positions could not be adequately determined using Mapviewer (NCBI, build 35.1) or NETAFFYX (Affymetrix, Santa Clara, CA).

### Nicotine dependence variants

In preplanned assessments of the allelic variants likely to influence vulnerability to dependence on nicotine and other addictive substances, we focused on autosomal SNPs that provided convergent data with four additional abuser *vs *comparisons datasets; *i.e. *SNPs that a) display t values with p < 0.005 nominal significance in comparisons between European-American controls *vs *nicotine dependent research participants; b) identify genes that also display reproducibly-positive associations with addiction vulnerabilities in data from four other samples: i) NIDA African-American and European-American polysubstance abuser *vs *control comparisons based on 639,401 SNP comparisons with the requirement that both samples provide nominally significant results (p < 0.0025 for the joint probability) and clustering so that at least three such SNPs lay within 100 Kb of each other [31] ii) JGIDA (Japanese genetic investigations of drug abuse) Japanese methamphetamine abuser *vs *control comparisons, based on a requirement for nominal significance (p < 0.05) of SNPs lying within the same genes [56] (*manuscript in preparation*) and iii) COGA (Collaborative study on the genetics of alcoholism) alcohol dependent *vs *control comparisons, based on a requirement for nominal significance (p < 0.05) of SNPs lying within the same genes [55] and c) produce an enhanced (*eg*. lower) Monte Carlo p value for the overall association in comparisons of the current smoker/control data with these four other sample sets *vs *the Monte Carlo p values for the data from the four other sample sets alone. Each of these Monte Carlo simulation trials began with sampling from a database that contains the results from the current study and results from a larger database that contains data from the prior association studies in the four additional samples noted above to which we compare the current results. For each of these 100,000 simulation trials, a randomly-selected set of SNPs was chosen and the same procedure that had been followed for the actual data was run. The number of trials for which the results from the randomly-selected set of SNPs matched or exceeded the results actually observed from the SNPs identified in the current study was tabulated. Empirical p values were calculated by dividing the number of trials for which the observed results were matched or exceeded by the total number of Monte Carlo simulation trials performed. Since this method examines the properties of the SNPs in the current dataset, assuming independence of their allele frequencies, it should be relatively robust despite the uneven distribution of Affymetrix SNP markers across the genome.

### Quit success variants

In comparing results related to successful abstinence, we use less stringent criteria. We focus on autosomal SNPs that display three features [see additional file 1]: 1) they display t values with p < 0.01 nominal significance in the current dataset of successful *vs *unsuccessful quitters; 2) they lie within clusters of at least three such nominally positive SNPs so that each positive SNP lies within 0.1 Mb of the nearest positive SNP; 3) they lie within genes whose functions can be inferred. We also compared these observed results to those expected by chance, based on independence of SNP allelic frequency estimates under the null hypothesis, using 10,000 – 100,000 Monte Carlo simulation trials on the database from the current study's results, as noted above [21].

### Statistical power

To assess the power of our current approach, we used the observed standard deviations and mean abuser/control differences for the SNPs that provided the largest differences between control and abuser population means, the program PS v2.1.31 [57] and α = 0.05.

### Control comparisons

To provide a control for the possibility that the abstainer/nonabstainer and user/control differences observed at some of the clustered, reproducibly-positive SNPs were due to occult ethnic/racial differences in the frequencies of alleles at these same SNPs between abstainers and non-abstainers or between abusers and controls, we compared the present results with those that we have previously obtained from comparisons of allele frequency data in self-reported African-American *vs *European-American control individuals, focusing on SNPs that display ethnicity difference scores that lie in the outlying +/- 2.5% of all differences (Table 1).

To provide a control for the possibility that the abuser-control differences observed at many of the clustered, reproducibly-positive SNPs were due to noisy assays for these SNPs, we examined the overlap between the clustered positive SNPs and the 2.5% of SNPs which display the largest variation between pools in data from this and other studies using the same arrays.

## Abreviations

DSM – diagnostic and statistical manual, CEPH – Center for human polymorphisms COGA – Collaborative study on the genetics of alcoholism, JGIDA – Japanese genetics initiative on drug abuse

## Authors' contributions

GRU conceived the study and initiated its key collaborations, designed the research, interpreted data, drafted and polished the manuscript

QRL synthesized hybridization probes from DNA pools and helped manage data

TD participated in DNA pool construction, array analysis, data management and manuscript preparation and polishing

CJ participated in data management and performed the statistical analysis

DW participated in DNA pool construction, array analyses and data management

JER oversaw the consenting and clinical assessments of subjects, participated in data analyses and interpretation and in manuscript preparation and polishing.

All authors read and approved the final manuscript

## Supplementary Material

Additional File 1 Successful-abstinence *vs *unsuccessful abstinence. Click here for file
