# Supplementary material for: Molecular genetics of nicotine dependence and abstinence: whole genome association using 520,000 SNPs
Source: BMC Genet. 2007 Apr 3;8:10. doi: 10.1186/1471-2156-8-10 (PMC1853105; doi:10.1186/1471-2156-8-10)
Supplement: Additional File 1 — Successful-abstinence vs unsuccessful abstinence. [file 1471-2156-8-10-S1.doc]

**Additional file 1:** Successful-abstinence *vs* unsuccessful abstinence

| ***SNP*** | ***chr*** | ***pos*** | ***gene*** | ***start_pos*** | ***Description*** |
| --- | --- | --- | --- | --- | --- |
| ***Cell adhesion axon guidance, extracellular matrix (n = 17)*** | | | | | |
| A-2022484 | 1 | 163,671,399 | GPA33 | 163,753,740 | glycoprotein A33 (transmembrane) |
| rs7545911 | 1 | 163,679,339 | GPA33 | 163,753,740 | glycoprotein A33 (transmembrane) |
| rs6682062 | 1 | 163,681,637 | GPA33 | 163,753,740 | glycoprotein A33 (transmembrane) |
| rs6688613 | 1 | 163,683,527 | GPA33 | 163,753,740 | glycoprotein A33 (transmembrane) |
| rs1327866 | 1 | 163,684,568 | GPA33 | 163,753,740 | glycoprotein A33 (transmembrane) |
| rs12133491 | 1 | 163,788,841 | GPA33 | 163,753,740 | glycoprotein A33 (transmembrane) |
| rs10127714 | 1 | 163,798,267 | GPA33 | 163,753,740 | glycoprotein A33 (transmembrane) |
| rs7412201 | 1 | 163,821,046 | GPA33 | 163,753,740 | glycoprotein A33 (transmembrane) |
| rs16858538 | 1 | 163,834,318 | GPA33 | 163,753,740 | glycoprotein A33 (transmembrane) |
| rs12143783 | 1 | 163,854,087 | GPA33 | 163,753,740 | glycoprotein A33 (transmembrane) |
| rs4652245 | 1 | 174,034,629 | KIAA1747 | 173,872,290 | astrotactin; protein from clone 24828 |
| rs4140633 | 1 | 174,036,597 | KIAA1747 | 173,872,290 | astrotactin; protein from clone 24828 |
| rs3946142 | 1 | 174,037,515 | KIAA1747 | 173,872,290 | astrotactin; protein from clone 24828 |
| rs6658794 | 1 | 174,045,855 | KIAA1747 | 173,872,290 | astrotactin; protein from clone 24828 |
| A-2047661 | 2 | 230,344,128 | DNER | 230,047,853 | delta-notch-like EGF repeat-containing transmembrane |
| rs12468084 | 2 | 230,421,857 | DNER | 230,047,853 | delta-notch-like EGF repeat-containing transmembrane |
| rs6733030 | 2 | 230,451,189 | DNER | 230,047,853 | delta-notch-like EGF repeat-containing transmembrane |
| rs927594 | 2 | 230,472,203 | DNER | 230,047,853 | delta-notch-like EGF repeat-containing transmembrane |
| rs1575468 | 2 | 230,480,187 | DNER | 230,047,853 | delta-notch-like EGF repeat-containing transmembrane |
| rs1526595 | 3 | 54,903,536 | HT017 | 54,927,423 | HT017 protein Leucine-rich repeats and transmembrane domains 2 (LRTM2) |
| rs9808962 | 3 | 54,916,481 | HT017 | 54,927,423 | HT017 protein Leucine-rich repeats and transmembrane domains 2 (LRTM2) |
| rs1918070 | 3 | 54,916,618 | HT017 | 54,927,423 | HT017 protein Leucine-rich repeats and transmembrane domains 2 (LRTM2) |
| rs2048805 | 3 | 54,946,579 | HT017 | 54,927,423 | HT017 protein Leucine-rich repeats and transmembrane domains 2 (LRTM2) |
| rs17054602 | 3 | 54,959,264 | HT017 | 54,927,423 | HT017 protein Leucine-rich repeats and transmembrane domains 2 (LRTM2) |
| rs3821659 | 3 | 54,962,963 | HT017 | 54,927,423 | HT017 protein Leucine-rich repeats and transmembrane domains 2 (LRTM2) |
| rs36179* | 3 | 136,129,270 | EPHB1 | 135,996,958 | EphB1 |
| rs36178* | 3 | 136,129,867 | EPHB1 | 135,996,958 | EphB1 |
| rs39704* | 3 | 136,129,905 | EPHB1 | 135,996,958 | EphB1 |
| rs40157 | 3 | 136,130,816 | EPHB1 | 135,996,958 | EphB1 |
| rs4955459 | 3 | 136,150,847 | EPHB1 | 135,996,958 | EphB1 |
| rs7644369 | 3 | 136,153,222 | EPHB1 | 135,996,958 | EphB1 |
| rs6785203 | 3 | 136,159,208 | EPHB1 | 135,996,958 | EphB1 |
| rs7699895 | 4 | 168,282,449 | SPOCK3 | 168,030,066 | sparc/osteonectin, cwcv and kazal-like domains proteoglycan (testican) 3 |
| rs7664643 | 4 | 168,319,485 | SPOCK3 | 168,030,066 | sparc/osteonectin, cwcv and kazal-like domains proteoglycan (testican) 3 |
| rs6818745 | 4 | 168,331,336 | SPOCK3 | 168,030,066 | sparc/osteonectin, cwcv and kazal-like domains proteoglycan (testican) 3 |
| rs6449781 | 5 | 64,643,239 | ADAMTS6 | 64,545,981 | a disintegrin-like and metalloprotease (reprolysin type) with thrombospondin type 1 motif, 6 |
| rs7705642 | 5 | 64,653,947 | ADAMTS6 | 64,545,981 | a disintegrin-like and metalloprotease (reprolysin type) with thrombospondin type 1 motif, 6 |
| rs2640717 | 5 | 64,676,668 | ADAMTS6 | 64,545,981 | a disintegrin-like and metalloprotease (reprolysin type) with thrombospondin type 1 motif, 6 |
| rs9291837 | 5 | 64,743,875 | ADAMTS6 | 64,545,981 | a disintegrin-like and metalloprotease (reprolysin type) with thrombospondin type 1 motif, 6 |
| rs2047064 | 5 | 64,769,683 | ADAMTS6 | 64,545,981 | a disintegrin-like and metalloprotease (reprolysin type) with thrombospondin type 1 motif, 6 |
| rs12659534 | 5 | 168,162,396 | SLIT3 | 168,025,857 | slit homolog 3 |
| rs1109106 | 5 | 168,177,820 | SLIT3 | 168,025,857 | slit homolog 3 |
| rs6897408 | 5 | 168,238,733 | SLIT3 | 168,025,857 | slit homolog 3 |
| rs1018631 | 6 | 40,379,505 | LRFN2 | 40,467,351 | leucine rich repeat and fibronectin type III domain containing 2 |
| rs4714329 | 6 | 40,381,435 | LRFN2 | 40,467,351 | leucine rich repeat and fibronectin type III domain containing 2 |
| rs715831 | 6 | 40,400,445 | LRFN2 | 40,467,351 | leucine rich repeat and fibronectin type III domain containing 2 |
| rs9462616 | 6 | 40,464,159 | LRFN2 | 40,467,351 | leucine rich repeat and fibronectin type III domain containing 2 |
| rs11154383 | 6 | 127,467,150 | THSD2 | 127,481,749 | thrombospondin, type I, domain containing 2 |
| rs2800708 | 6 | 127,479,310 | THSD2 | 127,481,749 | thrombospondin, type I, domain containing 2 |
| rs9482771 | 6 | 127,488,303 | THSD2 | 127,481,749 | thrombospondin, type I, domain containing 2 |
| rs2900132 | 9 | 116,489,192 | ASTN2 | 116,267,061 | astrotactin 2 |
| rs4837746 | 9 | 116,502,133 | ASTN2 | 116,267,061 | astrotactin 2 |
| rs10983314 | 9 | 116,554,626 | ASTN2 | 116,267,061 | astrotactin 2 |
| rs10983319 | 9 | 116,563,020 | ASTN2 | 116,267,061 | astrotactin 2 |
| rs2296869 | 9 | 132,201,966 | NTNG2 | 132,071,688 | netrin G2 |
| rs2296870* | 9 | 132,202,219 | NTNG2 | 132,071,688 | netrin G2 |
| A-2122450* | 9 | 132,203,239 | NTNG2 | 132,071,688 | netrin G2 |
| rs7868762 | 9 | 132,205,129 | NTNG2 | 132,071,688 | netrin G2 |
| rs7922905 | 10 | 20,534,950 | PLXDC2 | 20,145,378 | plexin domain containing 2 |
| rs7073927 | 10 | 20,556,872 | PLXDC2 | 20,145,378 | plexin domain containing 2 |
| rs7074175 | 10 | 20,556,984 | PLXDC2 | 20,145,378 | plexin domain containing 2 |
| rs7199326 | 16 | 81,818,112 | CDH13 | 81,218,079 | cadherin 13, H-cadherin |
| rs12933790 | 16 | 81,826,720 | CDH13 | 81,218,079 | cadherin 13, H-cadherin |
| rs2194341 | 16 | 81,837,945 | CDH13 | 81,218,079 | cadherin 13, H-cadherin |
| rs616019 | 18 | 7,733,140 | PTPRM | 7,557,817 | protein tyrosine phosphatase, receptor type, M |
| rs16952509 | 18 | 7,747,454 | PTPRM | 7,557,817 | protein tyrosine phosphatase, receptor type, M |
| rs727951 | 18 | 7,830,668 | PTPRM | 7,557,817 | protein tyrosine phosphatase, receptor type, M |
| rs6020113 | 20 | 35,736,459 | CTNNBL1 | 35,755,848 | catenin, beta like 1 |
| rs16986890 | 20 | 35,759,937 | CTNNBL1 | 35,755,848 | catenin, beta like 1 |
| rs6020846* | 20 | 35,839,081 | CTNNBL1 | 35,755,848 | catenin, beta like 1 |
| rs4809876 | 20 | 35,936,758 | CTNNBL1 | 35,755,848 | catenin, beta like 1 |
| rs6030070 | 20 | 40,292,402 | PTPRT | 40,134,807 | protein tyrosine phosphatase, receptor type, T |
| rs6030071 | 20 | 40,292,464 | PTPRT | 40,134,807 | protein tyrosine phosphatase, receptor type, T |
| rs6030076 | 20 | 40,296,596 | PTPRT | 40,134,807 | protein tyrosine phosphatase, receptor type, T |
| rs6030096 | 20 | 40,321,219 | PTPRT | 40,134,807 | protein tyrosine phosphatase, receptor type, T |
|  |  |  |  |  |  |
| ***Enzyme (n = 39)*** | | | | | |
| rs2744785 | 1 | 25,716,180 | MAN1C1 | 25,628,105 | mannosidase, alpha, class 1C, member 1 |
| rs807262 | 1 | 25,765,197 | MAN1C1 | 25,628,105 | mannosidase, alpha, class 1C, member 1 |
| rs807261 | 1 | 25,766,476 | MAN1C1 | 25,628,105 | mannosidase, alpha, class 1C, member 1 |
| rs2744785 | 1 | 25,716,180 | SEPN1 | 25,810,813 | selenoprotein N, 1 |
| rs807262 | 1 | 25,765,197 | SEPN1 | 25,810,813 | selenoprotein N, 1 |
| rs807261 | 1 | 25,766,476 | SEPN1 | 25,810,813 | selenoprotein N, 1 |
| rs785109 | 1 | 39,822,489 | PPIE | 39,873,623 | peptidylprolyl isomerase E (cyclophilin E) |
| rs914961 | 1 | 39,911,538 | PPIE | 39,873,623 | peptidylprolyl isomerase E (cyclophilin E) |
| rs1974206 | 1 | 39,978,011 | PPIE | 39,873,623 | peptidylprolyl isomerase E (cyclophilin E) |
| rs785109 | 1 | 39,822,489 | BMP8B | 39,892,996 | bone morphogenetic protein 8b (osteogenic protein 2) |
| rs914961 | 1 | 39,911,538 | BMP8B | 39,892,996 | bone morphogenetic protein 8b (osteogenic protein 2) |
| rs1974206 | 1 | 39,978,011 | BMP8B | 39,892,996 | bone morphogenetic protein 8b (osteogenic protein 2) |
| rs785109 | 1 | 39,822,489 | OXCT2 | 39,904,290 | 3-oxoacid CoA transferase 2 |
| rs914961 | 1 | 39,911,538 | OXCT2 | 39,904,290 | 3-oxoacid CoA transferase 2 |
| rs1974206 | 1 | 39,978,011 | OXCT2 | 39,904,290 | 3-oxoacid CoA transferase 2 |
| rs11264275 | 1 | 151,638,343 | PMVK | 151,710,283 | phosphomevalonate kinase |
| rs10908448 | 1 | 151,695,617 | PMVK | 151,710,283 | phosphomevalonate kinase |
| rs4845690 | 1 | 151,699,890 | PMVK | 151,710,283 | phosphomevalonate kinase |
| rs17557525 | 1 | 220,214,033 | CAPN2 | 220,206,936 | calpain 2, (m/II) large subunit |
| rs12088254 | 1 | 220,274,086 | CAPN2 | 220,206,936 | calpain 2, (m/II) large subunit |
| rs6678365 | 1 | 220,330,469 | CAPN2 | 220,206,936 | calpain 2, (m/II) large subunit |
| rs17037369 | 3 | 38,103,093 | ACAA1 | 38,139,211 | acetyl-Coenzyme A acyltransferase 1 (peroxisomal 3-oxoacyl-Coenzyme A thiolase) |
| rs6599263* | 3 | 38,119,879 | ACAA1 | 38,139,211 | acetyl-Coenzyme A acyltransferase 1 (peroxisomal 3-oxoacyl-Coenzyme A thiolase) |
| rs6767684 | 3 | 38,160,178 | ACAA1 | 38,139,211 | acetyl-Coenzyme A acyltransferase 1 (peroxisomal 3-oxoacyl-Coenzyme A thiolase) |
| rs17037369 | 3 | 38,103,093 | OSR1 | 38,182,030 | oxidative-stress responsive 1 Ser/Thr protein kinase |
| rs6599263* | 3 | 38,119,879 | OSR1 | 38,182,030 | oxidative-stress responsive 1 Ser/Thr protein kinase |
| rs6767684 | 3 | 38,160,178 | OSR1 | 38,182,030 | oxidative-stress responsive 1 Ser/Thr protein kinase |
| rs3772891 | 3 | 81,859,450 | GBE1 | 81,621,548 | glucan (1,4-alpha-), branching enzyme 1 |
| rs1464790 | 3 | 81,935,613 | GBE1 | 81,621,548 | glucan (1,4-alpha-), branching enzyme 1 |
| rs1524557 | 3 | 81,974,721 | GBE1 | 81,621,548 | glucan (1,4-alpha-), branching enzyme 1 |
| rs1464791 | 3 | 81,976,619 | GBE1 | 81,621,548 | glucan (1,4-alpha-), branching enzyme 1 ( |
| rs9836487* | 3 | 141,660,977 | CLSTN2 | 141,136,905 | calsyntenin 2 |
| rs4683499 | 3 | 141,662,928 | CLSTN2 | 141,136,905 | calsyntenin 2 |
| rs349504* | 3 | 141,712,143 | CLSTN2 | 141,136,905 | calsyntenin 2 |
| rs16850556 | 3 | 141,772,839 | CLSTN2 | 141,136,905 | calsyntenin 2 |
| rs13103430 | 4 | 17,243,624 | QDPR | 17,164,292 | quinoid dihydropteridine reductase |
| rs3733576 | 4 | 17,262,972 | QDPR | 17,164,292 | quinoid dihydropteridine reductase |
| rs6449314 | 4 | 17,264,706 | QDPR | 17,164,292 | quinoid dihydropteridine reductase |
| rs4698629 | 4 | 17,285,153 | QDPR | 17,164,292 | quinoid dihydropteridine reductase |
| rs13103430 | 4 | 17,243,624 | LAP3 | 17,255,196 | leucine aminopeptidase 3 |
| rs3733576 | 4 | 17,262,972 | LAP3 | 17,255,196 | leucine aminopeptidase 3 |
| rs6449314 | 4 | 17,264,706 | LAP3 | 17,255,196 | leucine aminopeptidase 3 |
| rs4698629 | 4 | 17,285,153 | LAP3 | 17,255,196 | leucine aminopeptidase 3 |
| rs4698634* | 4 | 17,306,461 | LAP3 | 17,255,196 | leucine aminopeptidase 3 |
| rs10061370* | 5 | 146,048,554 | PPP2R2B | 145,949,260 | protein phosphatase 2 (formerly 2A), regulatory subunit B (PR 52), beta isoform |
| rs2400228* | 5 | 146,049,791 | PPP2R2B | 145,949,260 | protein phosphatase 2 (formerly 2A), regulatory subunit B (PR 52), beta isoform |
| rs7716683* | 5 | 146,060,205 | PPP2R2B | 145,949,260 | protein phosphatase 2 (formerly 2A), regulatory subunit B (PR 52), beta isoform |
| rs17106458* | 5 | 146,693,780 | STK32A | 146,594,862 | serine/threonine kinase 32A |
| rs10079191 | 5 | 146,696,881 | STK32A | 146,594,862 | serine/threonine kinase 32A |
| rs6884181 | 5 | 146,703,083 | STK32A | 146,594,862 | serine/threonine kinase 32A |
| rs918797 | 5 | 146,708,683 | STK32A | 146,594,862 | serine/threonine kinase 32A |
| rs4705166 | 5 | 146,709,417 | STK32A | 146,594,862 | serine/threonine kinase 32A |
| rs2288806 | 5 | 146,755,668 | STK32A | 146,594,862 | serine/threonine kinase 32A |
| rs17550470 | 5 | 146,759,060 | STK32A | 146,594,862 | serine/threonine kinase 32A |
| rs12655364 | 5 | 146,759,414 | STK32A | 146,594,862 | serine/threonine kinase 32A |
| rs3805536* | 5 | 146,763,849 | STK32A | 146,594,862 | serine/threonine kinase 32A |
| rs17106458* | 5 | 146,693,780 | DPYSL3 | 146,750,566 | dihydropyrimidinase-like 3 |
| rs10079191 | 5 | 146,696,881 | DPYSL3 | 146,750,566 | dihydropyrimidinase-like 3 |
| rs6884181* | 5 | 146,703,083 | DPYSL3 | 146,750,566 | dihydropyrimidinase-like 3 |
| rs918797 | 5 | 146,708,683 | DPYSL3 | 146,750,566 | dihydropyrimidinase-like 3 |
| rs4705166 | 5 | 146,709,417 | DPYSL3 | 146,750,566 | dihydropyrimidinase-like 3 |
| rs2288806 | 5 | 146,755,668 | DPYSL3 | 146,750,566 | dihydropyrimidinase-like 3 |
| rs17550470 | 5 | 146,759,060 | DPYSL3 | 146,750,566 | dihydropyrimidinase-like 3 |
| rs12655364 | 5 | 146,759,414 | DPYSL3 | 146,750,566 | dihydropyrimidinase-like 3 |
| rs3805536* | 5 | 146,763,849 | DPYSL3 | 146,750,566 | dihydropyrimidinase-like 3 |
| rs13160107 | 5 | 76,753,339 | PDE8B | 76,542,462 | phosphodiesterase 8B |
| rs335628 | 5 | 76,765,294 | PDE8B | 76,542,462 | phosphodiesterase 8B |
| rs3797642 | 5 | 76,784,682 | PDE8B | 76,542,462 | phosphodiesterase 8B |
| rs9366344 | 6 | 20,435,828 | OACT1 | 20,208,928 | O-acyltransferase (membrane bound) domain containing 1 |
| rs9358331 | 6 | 20,452,975 | OACT1 | 20,208,928 | O-acyltransferase (membrane bound) domain containing 1 |
| rs9356730 | 6 | 20,453,095 | OACT1 | 20,208,928 | O-acyltransferase (membrane bound) domain containing 1 |
| rs17156320 | 7 | 76,944,761 | PTPN12 | 76,811,486 | protein tyrosine phosphatase, non-receptor type 12 |
| rs17807185 | 7 | 76,952,946 | PTPN12 | 76,811,486 | protein tyrosine phosphatase, non-receptor type 12 |
| rs12113119 | 7 | 76,959,469 | PTPN12 | 76,811,486 | protein tyrosine phosphatase, non-receptor type 12 |
| rs17128267 | 8 | 19,257,352 | ChGn | 19,305,952 | chondroitin beta1,4 N-acetylgalactosaminyltransferase |
| rs17128272 | 8 | 19,257,994 | ChGn | 19,305,952 | chondroitin beta1,4 N-acetylgalactosaminyltransferase |
| rs6586833 | 8 | 19,350,542 | ChGn | 19,305,952 | chondroitin beta1,4 N-acetylgalactosaminyltransferase |
| rs2137157 | 10 | 23,765,898 | OTUD1 | 23,768,393 | OTU domain containing 1 |
| rs4747475 | 10 | 23,783,468 | OTUD1 | 23,768,393 | OTU domain containing 1 |
| rs6482286* | 10 | 23,808,903 | OTUD1 | 23,768,393 | OTU domain containing 1 |
| rs2816836 | 10 | 52,422,379 | PRKG1 | 52,504,299 | protein kinase, cGMP-dependent, type I |
| rs2816835 | 10 | 52,422,640 | PRKG1 | 52,504,299 | protein kinase, cGMP-dependent, type I |
| rs7069183 | 10 | 52,426,345 | PRKG1 | 52,504,299 | protein kinase, cGMP-dependent, type I |
| rs10995339 | 10 | 52,437,180 | PRKG1 | 52,504,299 | protein kinase, cGMP-dependent, type I |
| rs7895755 | 10 | 52,444,054 | PRKG1 | 52,504,299 | protein kinase, cGMP-dependent, type I |
| rs10822095 | 10 | 52,444,923 | PRKG1 | 52,504,299 | protein kinase, cGMP-dependent, type I |
| rs9299465 | 10 | 52,544,506 | PRKG1 | 52,504,299 | protein kinase, cGMP-dependent, type I |
| rs629119 | 11 | 93,890,094 | FUT4 | 93,916,775 | fucosyltransferase 4 (alpha (1,3) fucosyltransferase, myeloid-specific) |
| rs629156 | 11 | 93,890,123 | FUT4 | 93,916,775 | fucosyltransferase 4 (alpha (1,3) fucosyltransferase, myeloid-specific) |
| rs16924603 | 11 | 93,894,907 | FUT4 | 93,916,775 | fucosyltransferase 4 (alpha (1,3) fucosyltransferase, myeloid-specific) |
| rs11020812 | 11 | 93,906,765 | FUT4 | 93,916,775 | fucosyltransferase 4 (alpha (1,3) fucosyltransferase, myeloid-specific) |
| rs11020836 | 11 | 93,948,540 | FUT4 | 93,916,775 | fucosyltransferase 4 (alpha (1,3) fucosyltransferase, myeloid-specific) |
| rs948379 | 11 | 120,625,340 | SC5DL | 120,679,285 | sterol-C5-desaturase (ERG3 delta-5-desaturase homolog, fungal)-like |
| rs10892714 | 11 | 120,642,900 | SC5DL | 120,679,285 | sterol-C5-desaturase (ERG3 delta-5-desaturase homolog, fungal)-like |
| rs1583124 | 11 | 120,660,151 | SC5DL | 120,679,285 | sterol-C5-desaturase (ERG3 delta-5-desaturase homolog, fungal)-like |
| rs1469535 | 11 | 120,738,796 | SC5DL | 120,679,285 | sterol-C5-desaturase (ERG3 delta-5-desaturase homolog, fungal)-like |
| rs2267873 | 14 | 79,743,567 | DIO2 | 79,733,626 | deiodinase, iodothyronine, type II |
| rs2295138 | 14 | 79,813,376 | DIO2 | 79,733,626 | deiodinase, iodothyronine, type II |
| rs9989235 | 14 | 79,837,480 | DIO2 | 79,733,626 | deiodinase, iodothyronine, type II |
| rs2160355 | 14 | 79,841,000 | DIO2 | 79,733,626 | deiodinase, iodothyronine, type II |
| rs11628803 | 14 | 92,140,517 | LGMN | 92,239,907 | legumain cysteine protease with specificity for hydrolylzing asparaginyl bonds |
| rs2146498 | 14 | 92,142,022 | LGMN | 92,239,907 | legumain cysteine protease with specificity for hydrolylzing asparaginyl bonds |
| rs4904964 | 14 | 92,169,620 | LGMN | 92,239,907 | legumain cysteine protease with specificity for hydrolylzing asparaginyl bonds |
| rs2753934 | 14 | 93,921,083 | SERPINA1 | 93,914,463 | serine (or cysteine) proteinase inhibitor, clade A (alpha-1 antitrypsin), member 1 |
| rs8008729 | 14 | 94,019,874 | SERPINA1 | 93,914,463 | serine (or cysteine) proteinase inhibitor, clade A (alpha-1 antitrypsin), member 1 |
| rs12589932 | 14 | 94,020,846 | SERPINA1 | 93,914,463 | serine (or cysteine) proteinase inhibitor, clade A (alpha-1 antitrypsin), member 1 |
| rs2753934 | 14 | 93,921,083 | SERPINA11 | 93,978,556 | serine (or cysteine) proteinase inhibitor, clade A (alpha-1 antitrypsin), member 11 |
| rs8008729 | 14 | 94,019,874 | SERPINA11 | 93,978,556 | serine (or cysteine) proteinase inhibitor, clade A (alpha-1 antitrypsin), member 11 |
| rs12589932 | 14 | 94,020,846 | SERPINA11 | 93,978,556 | serine (or cysteine) proteinase inhibitor, clade A (alpha-1 antitrypsin), member 11 |
| rs4900233 | 14 | 94,026,811 | SERPINA11 | 93,978,556 | serine (or cysteine) proteinase inhibitor, clade A (alpha-1 antitrypsin), member 11 |
| rs8012043 | 14 | 94,033,174 | SERPINA11 | 93,978,556 | serine (or cysteine) proteinase inhibitor, clade A (alpha-1 antitrypsin), member 11 |
| rs2753934 | 14 | 93,921,083 | SERPINA9 | 94,000,716 | serine (or cysteine) proteinase inhibitor, clade A (alpha-1 antitrypsin), member 9 |
| rs8008729 | 14 | 94,019,874 | SERPINA9 | 94,000,716 | serine (or cysteine) proteinase inhibitor, clade A (alpha-1 antitrypsin), member 9 |
| rs12589932 | 14 | 94,020,846 | SERPINA9 | 94,000,716 | serine (or cysteine) proteinase inhibitor, clade A (alpha-1 antitrypsin), member 9 |
| rs4900233 | 14 | 94,026,811 | SERPINA9 | 94,000,716 | serine (or cysteine) proteinase inhibitor, clade A (alpha-1 antitrypsin), member 9 |
| rs8012043 | 14 | 94,033,174 | SERPINA9 | 94,000,716 | serine (or cysteine) proteinase inhibitor, clade A (alpha-1 antitrypsin), member 9 |
| rs8008729 | 14 | 94,019,874 | SERPINA12 | 94,023,374 | serine (or cysteine) proteinase inhibitor, clade A (alpha-1 antitrypsin), member 12 |
| rs12589932 | 14 | 94,020,846 | SERPINA12 | 94,023,374 | serine (or cysteine) proteinase inhibitor, clade A (alpha-1 antitrypsin), member 12 |
| rs4900233 | 14 | 94,026,811 | SERPINA12 | 94,023,374 | serine (or cysteine) proteinase inhibitor, clade A (alpha-1 antitrypsin), member 12 |
| rs8012043 | 14 | 94,033,174 | SERPINA12 | 94,023,374 | serine (or cysteine) proteinase inhibitor, clade A (alpha-1 antitrypsin), member 12 |
| rs8008729 | 14 | 94,019,874 | SERPINA4 | 94,097,536 | serine (or cysteine) proteinase inhibitor, clade A (alpha-1 antitrypsin), member 4 |
| rs12589932 | 14 | 94,020,846 | SERPINA4 | 94,097,536 | serine (or cysteine) proteinase inhibitor, clade A (alpha-1 antitrypsin), member 4 |
| rs4900233 | 14 | 94,026,811 | SERPINA4 | 94,097,536 | serine (or cysteine) proteinase inhibitor, clade A (alpha-1 antitrypsin), member 4 |
| rs8012043 | 14 | 94,033,174 | SERPINA4 | 94,097,536 | serine (or cysteine) proteinase inhibitor, clade A (alpha-1antitrypsin), member 4 |
| rs8008729 | 14 | 94,019,874 | SERPINA5 | 94,117,564 | serine (or cysteine) proteinase inhibitor, clade A (alpha-1 antitrypsin), member 5 |
| rs12589932 | 14 | 94,020,846 | SERPINA5 | 94,117,564 | serine (or cysteine) proteinase inhibitor, clade A (alpha-1 antitrypsin), member 5 |
| rs4900233 | 14 | 94,026,811 | SERPINA5 | 94,117,564 | serine (or cysteine) proteinase inhibitor, clade A (alpha-1 antitrypsin), member 5 |
| rs8012043 | 14 | 94,033,174 | SERPINA5 | 94,117,564 | serine (or cysteine) proteinase inhibitor, clade A (alpha-1 antitrypsin), member 5 |
| rs2925253 | 15 | 23,427,097 | ATP10A | 23,473,513 | ATPase, Class V, type 10A |
| rs4906777 | 15 | 23,600,150 | ATP10A | 23,473,513 | ATPase, Class V, type 10A |
| rs17642892 | 15 | 23,604,395 | ATP10A | 23,473,513 | ATPase, Class V, type 10A |
| rs8041836 | 15 | 23,605,988 | ATP10A | 23,473,513 | ATPase, Class V, type 10A |
| rs12444183 | 16 | 80,330,710 | PLCG2 | 80,370,431 | phospholipase C, gamma 2 (phosphatidylinositol-specific) |
| rs6564919 | 16 | 80,374,081 | PLCG2 | 80,370,431 | phospholipase C, gamma 2 (phosphatidylinositol-specific) |
| rs7192802 | 16 | 80,374,523 | PLCG2 | 80,370,431 | phospholipase C, gamma 2 (phosphatidylinositol-specific) |
| rs4580153 | 16 | 80,374,740 | PLCG2 | 80,370,431 | phospholipase C, gamma 2 (phosphatidylinositol-specific) |
| rs11079856 | 17 | 44,545,771 | GALGT2 | 44,565,328 | UDP-GalNAc:Neu5Acalpha2-3Galbeta-R beta1,4-N-acetylgalactosaminyltransferase |
| rs1554551 | 17 | 44,548,140 | GALGT2 | 44,565,328 | UDP-GalNAc:Neu5Acalpha2-3Galbeta-R beta1,4-N-acetylgalactosaminyltransferase |
| rs17636326 | 17 | 44,572,905 | GALGT2 | 44,565,328 | UDP-GalNAc:Neu5Acalpha2-3Galbeta-R beta1,4-N-acetylgalactosaminyltransferase |
| rs11079856 | 17 | 44,545,771 | GNGT2 | 44,638,596 | guanine nucleotide binding protein (G protein), gamma transducing activity polypeptide 2 |
| rs1554551 | 17 | 44,548,140 | GNGT2 | 44,638,596 | guanine nucleotide binding protein (G protein), gamma transducing activity polypeptide 2 |
| rs17636326 | 17 | 44,572,905 | GNGT2 | 44,638,596 | guanine nucleotide binding protein (G protein), gamma transducing activity polypeptide 2 |
| rs12460892 | 19 | 34,456,548 | UQCRFS1 | 34,390,007 | ubiquinol-cytochrome c reductase, Rieske iron-sulfur polypeptide 1 |
| rs6509333 | 19 | 34,468,249 | UQCRFS1 | 34,390,007 | ubiquinol-cytochrome c reductase, Rieske iron-sulfur polypeptide 1 |
| rs7260650 | 19 | 34,469,167 | UQCRFS1 | 34,390,007 | ubiquinol-cytochrome c reductase, Rieske iron-sulfur polypeptide 1 |
| rs759632 | 19 | 34,472,578 | UQCRFS1 | 34,390,007 | ubiquinol-cytochrome c reductase, Rieske iron-sulfur polypeptide 1 |
| rs10406799 | 19 | 34,475,403 | UQCRFS1 | 34,390,007 | ubiquinol-cytochrome c reductase, Rieske iron-sulfur polypeptide 1 |
| rs11083894 | 19 | 34,476,307 | UQCRFS1 | 34,390,007 | ubiquinol-cytochrome c reductase, Rieske iron-sulfur polypeptide 1 |
| rs17624382 | 19 | 34,480,470 | UQCRFS1 | 34,390,007 | ubiquinol-cytochrome c reductase, Rieske iron-sulfur polypeptide 1 |
| rs1048546 | 21 | 29,166,748 | C21ORF127 | 29,170,347 | HemK methyltransferase family member 2 |
| rs2254638 | 21 | 29,178,154 | C21ORF127 | 29,170,347 | HemK methyltransferase family member 2 |
| rs2248501 | 21 | 29,198,282 | C21ORF127 | 29,170,347 | HemK methyltransferase family member 2 |
| rs2832137* | 21 | 29,201,887 | C21ORF127 | 29,170,347 | HemK methyltransferase family member 2 |
| rs2832147 | 21 | 29,266,813 | C21ORF127 | 29,170,347 | HemK methyltransferase family member 2 |
| rs4820483 | 22 | 41,512,678 | ARFGAP3 | 41,517,030 | ADP-ribosylation factor GTPase activating protein 3 |
| rs5758965 | 22 | 41,551,097 | ARFGAP3 | 41,517,030 | ADP-ribosylation factor GTPase activating protein 3 |
| rs738537 | 22 | 41,602,420 | ARFGAP3 | 41,517,030 | ADP-ribosylation factor GTPase activating protein 3 |
| rs12166809 | 22 | 41,611,788 | ARFGAP3 | 41,517,030 | ADP-ribosylation factor GTPase activating protein 3 |
| rs4820494 | 22 | 41,630,359 | ARFGAP3 | 41,517,030 | ADP-ribosylation factor GTPase activating protein 3 |
| rs1071960 | 22 | 41,674,130 | ARFGAP3 | 41,517,030 | ADP-ribosylation factor GTPase activating protein 3 |
|  |  |  |  |  |  |
| ***Receptor, ligand, GTPase (n = 37)*** | | | | | |
| rs1171262 | 1 | 65,713,767 | LEPR | 65,603,007 | leptin receptor |
| rs3790432 | 1 | 65,747,760 | LEPR | 65,603,007 | leptin receptor |
| rs3790426 | 1 | 65,755,040 | LEPR | 65,603,007 | leptin receptor |
| rs17125608 | 1 | 102,039,164 | OLFM3 | 101,980,154 | olfactomedin 3 |
| rs7515984 | 1 | 102,107,241 | OLFM3 | 101,980,154 | olfactomedin 3 |
| rs755181 | 1 | 102,116,268 | OLFM3 | 101,980,154 | olfactomedin 3 |
| rs7535852 | 1 | 102,116,304 | OLFM3 | 101,980,154 | olfactomedin 3 |
| rs1887467 | 1 | 102,119,334 | OLFM3 | 101,980,154 | olfactomedin 3 |
| rs7512461 | 1 | 102,120,783 | OLFM3 | 101,980,154 | olfactomedin 3 |
| rs11583344 | 1 | 102,123,899 | OLFM3 | 101,980,154 | olfactomedin 3 |
| rs2044470 | 2 | 174,757,344 | PTD004 | 174,762,684 | PTD004 GTP binding protein |
| rs10930638 | 2 | 174,800,103 | PTD004 | 174,762,684 | PTD004 GTP binding protein |
| rs17239377 | 2 | 174,805,846 | PTD004 | 174,762,684 | PTD004 GTP binding protein |
| rs17037369 | 3 | 38,103,093 | MYD88 | 38,155,157 | myeloid differentiation primary response gene (88) |
| rs6599263* | 3 | 38,119,879 | MYD88 | 38,155,157 | myeloid differentiation primary response gene (88) |
| rs6767684 | 3 | 38,160,178 | MYD88 | 38,155,157 | myeloid differentiation primary response gene (88) |
| rs876535 | 3 | 68,923,672 | TAFA4 | 68,863,607 | TAFA4 Family sequence similarity 19 (chemokine (C-C motif)-like), member A4 (FAM19A4) |
| rs4855516 | 3 | 68,940,970 | TAFA4 | 68,863,607 | TAFA4 Family sequence similarity 19 (chemokine (C-C motif)-like), member A4 (FAM19A4) |
| rs2130439 | 3 | 68,952,118 | TAFA4 | 68,863,607 | TAFA4 Family sequence similarity 19 (chemokine (C-C motif)-like), member A4 (FAM19A4) |
| rs10470577 | 3 | 68,956,675 | TAFA4 | 68,863,607 | TAFA4 Family sequence similarity 19 (chemokine (C-C motif)-like), member A4 (FAM19A4) |
| rs2102860 | 3 | 188,593,685 | IFRG28 | 188,568,878 | 28kD interferon responsive protein RTP4 receptor transporter protein 4 |
| rs11924176 | 3 | 188,635,585 | IFRG28 | 188,568,878 | 28kD interferon responsive protein RTP4 receptor transporter protein 4 |
| rs2687926 | 3 | 188,636,568 | IFRG28 | 188,568,878 | 28kD interferon responsive protein RTP4 receptor transporter protein 4 |
| rs3860110 | 5 | 134,896,238 | CXCL14 | 134,934,274 | chemokine (C-X-C motif) ligand 14 |
| rs2344485 | 5 | 134,896,540 | CXCL14 | 134,934,274 | chemokine (C-X-C motif) ligand 14 |
| rs11739936 | 5 | 134,941,470 | CXCL14 | 134,934,274 | chemokine (C-X-C motif) ligand 14 |
| rs17168737 | 5 | 134,971,400 | CXCL14 | 134,934,274 | chemokine (C-X-C motif) ligand 14 |
| rs4710183 | 6 | 167,484,494 | FGFR1OP | 167,383,227 | FGFR1 oncogene partner |
| rs975822* | 6 | 167,486,869 | FGFR1OP | 167,383,227 | FGFR1 oncogene partner |
| rs6907666 | 6 | 167,493,806 | FGFR1OP | 167,383,227 | FGFR1 oncogene partner |
| rs2021033 | 6 | 167,494,650 | FGFR1OP | 167,383,227 | FGFR1 oncogene partner |
| rs6456158 | 6 | 167,498,443 | FGFR1OP | 167,383,227 | FGFR1 oncogene partner |
| rs4710183 | 6 | 167,484,494 | CCR6 | 167,506,668 | chemokine (C-C motif) receptor 6 |
| rs975822* | 6 | 167,486,869 | CCR6 | 167,506,668 | chemokine (C-C motif) receptor 6 |
| rs6907666 | 6 | 167,493,806 | CCR6 | 167,506,668 | chemokine (C-C motif) receptor 6 |
| rs2021033 | 6 | 167,494,650 | CCR6 | 167,506,668 | chemokine (C-C motif) receptor 6 |
| rs6456158 | 6 | 167,498,443 | CCR6 | 167,506,668 | chemokine (C-C motif) receptor 6 |
| rs4710183 | 6 | 167,484,494 | GPR31 | 167,540,771 | G protein-coupled receptor 31 |
| rs975822* | 6 | 167,486,869 | GPR31 | 167,540,771 | G protein-coupled receptor 31 |
| rs6907666 | 6 | 167,493,806 | GPR31 | 167,540,771 | G protein-coupled receptor 31 |
| rs2021033 | 6 | 167,494,650 | GPR31 | 167,540,771 | G protein-coupled receptor 31 |
| rs6456158 | 6 | 167,498,443 | GPR31 | 167,540,771 | G protein-coupled receptor 31 |
| rs17597696 | 2 | 33,687,316 | RASGRP3 | 33,573,067 | RAS guanyl releasing protein 3 (calcium and DAG-regulated) |
| rs17013337 | 2 | 33,694,732 | RASGRP3 | 33,573,067 | RAS guanyl releasing protein 3 (calcium and DAG-regulated) |
| rs897506 | 2 | 33,698,163 | RASGRP3 | 33,573,067 | RAS guanyl releasing protein 3 (calcium and DAG-regulated) |
| rs2970990 | 2 | 100,658,947 | PDCL3 | 100,637,957 | phosducin-like 3 |
| rs2309846 | 2 | 100,659,966 | PDCL3 | 100,637,957 | phosducin-like 3 |
| rs4851365 | 2 | 100,749,655 | PDCL3 | 100,637,957 | phosducin-like 3 |
| rs12468084 | 2 | 230,421,857 | TRIP12 | 230,457,435 | thyroid hormone receptor interactor 12 |
| rs6733030 | 2 | 230,451,189 | TRIP12 | 230,457,435 | thyroid hormone receptor interactor 12 |
| rs927594 | 2 | 230,472,203 | TRIP12 | 230,457,435 | thyroid hormone receptor interactor 12 |
| rs1575468 | 2 | 230,480,187 | TRIP12 | 230,457,435 | thyroid hormone receptor interactor 12 |
| rs546530 | 2 | 230,578,469 | TRIP12 | 230,457,435 | thyroid hormone receptor interactor 12 |
| rs6757156 | 2 | 230,646,105 | TRIP12 | 230,457,435 | thyroid hormone receptor interactor 12 |
| rs486012 | 3 | 9,016,299 | SRGAP2 | 8,997,278 | SLIT-ROBO Rho GTPase activating protein 2 |
| rs341795 | 3 | 9,021,906 | SRGAP2 | 8,997,278 | SLIT-ROBO Rho GTPase activating protein 2 |
| rs884235 | 3 | 9,054,481 | SRGAP2 | 8,997,278 | SLIT-ROBO Rho GTPase activating protein 2 |
| rs2669986 | 3 | 9,098,116 | SRGAP2 | 8,997,278 | SLIT-ROBO Rho GTPase activating protein 2 |
| rs531719 | 3 | 9,111,060 | SRGAP2 | 8,997,278 | SLIT-ROBO Rho GTPase activating protein 2 |
| rs6765880 | 3 | 9,207,109 | SRGAP2 | 8,997,278 | SLIT-ROBO Rho GTPase activating protein 2 |
| rs6969223 | 7 | 44,828,454 | TBRG4 | 44,912,940 | transforming growth factor beta regulator 4 |
| rs1294966 | 7 | 44,847,039 | TBRG4 | 44,912,940 | transforming growth factor beta regulator 4 |
| rs3735486 | 7 | 44,878,357 | TBRG4 | 44,912,940 | transforming growth factor beta regulator 4 |
| rs3757572 | 7 | 44,919,896 | TBRG4 | 44,912,940 | transforming growth factor beta regulator 4 |
| rs10224439* | 7 | 44,954,161 | TBRG4 | 44,912,940 | transforming growth factor beta regulator 4 |
| rs3735486 | 7 | 44,878,357 | RAMP3 | 44,970,639 | receptor (calcitonin) activity modifying protein 3 |
| rs3757572 | 7 | 44,919,896 | RAMP3 | 44,970,639 | receptor (calcitonin) activity modifying protein 3 |
| rs10224439* | 7 | 44,954,161 | RAMP3 | 44,970,639 | receptor (calcitonin) activity modifying protein 3 |
| rs7810209 | 7 | 149,591,461 | GIMAP8 | 149,585,772 | human immune associated nucleotide 6 GTPase, IMAP family member 8 |
| rs10952266 | 7 | 149,668,340 | GIMAP8 | 149,585,772 | human immune associated nucleotide 6 GTPase, IMAP family member 8 |
| rs13442834 | 7 | 149,682,904 | GIMAP8 | 149,585,772 | human immune associated nucleotide 6 GTPase, IMAP family member 8 |
| rs7810209 | 7 | 149,591,461 | GIMAP7 | 149,649,611 | immune associated nucleotide 6 GTPase, IMAP family member 7 |
| rs10952266 | 7 | 149,668,340 | GIMAP7 | 149,649,611 | immune associated nucleotide 6 GTPase, IMAP family member 7 |
| rs13442834 | 7 | 149,682,904 | GIMAP7 | 149,649,611 | immune associated nucleotide 6 GTPase, IMAP family member 7 |
| A-2049472 | 8 | 67,189,136 | CRH | 67,251,173 | corticotropin releasing hormone |
| rs7831234 | 8 | 67,214,949 | CRH | 67,251,173 | corticotropin releasing hormone |
| rs9694082* | 8 | 67,229,334 | CRH | 67,251,173 | corticotropin releasing hormone |
| rs7822243* | 8 | 67,231,942 | CRH | 67,251,173 | corticotropin releasing hormone |
| rs6999100* | 8 | 67,245,286 | CRH | 67,251,173 | corticotropin releasing hormone |
| rs984430 | 9 | 84,812,381 | NTRK2 | 84,514,247 | neurotrophic tyrosine kinase, receptor, type 2 |
| rs4877895 | 9 | 84,842,877 | NTRK2 | 84,514,247 | neurotrophic tyrosine kinase, receptor, type 2 |
| rs4242632 | 9 | 84,848,765 | NTRK2 | 84,514,247 | neurotrophic tyrosine kinase, receptor, type 2 |
| rs1565450 | 9 | 84,880,389 | NTRK2 | 84,514,247 | neurotrophic tyrosine kinase, receptor, type 2 |
| rs2780936 | 9 | 84,929,200 | NTRK2 | 84,514,247 | neurotrophic tyrosine kinase, receptor, type 2 |
| rs866624 | 9 | 111,167,251 | OR2K2 | 111,169,318 | olfactory receptor, family 2, subfamily K, member 2 |
| rs6477821 | 9 | 111,224,584 | OR2K2 | 111,169,318 | olfactory receptor, family 2, subfamily K, member 2 |
| rs7390341 | 9 | 111,242,812 | OR2K2 | 111,169,318 | olfactory receptor, family 2, subfamily K, member 2 |
| rs12350676 | 9 | 111,249,685 | OR2K2 | 111,169,318 | olfactory receptor, family 2, subfamily K, member 2 |
| rs3765543 | 9 | 131,487,878 | RAPGEF1 | 131,481,711 | Rap guanine nucleotide exchange factor (GEF) 1 |
| rs2282009 | 9 | 131,525,523 | RAPGEF1 | 131,481,711 | Rap guanine nucleotide exchange factor (GEF) 1 |
| rs6597515 | 9 | 131,561,966 | RAPGEF1 | 131,481,711 | Rap guanine nucleotide exchange factor (GEF) 1 |
| rs9943519 | 11 | 55,235,453 | OR4C6 | 55,189,216 | olfactory receptor, family 4, subfamily C, member 6 |
| rs2127230 | 11 | 55,253,465 | OR4C6 | 55,189,216 | olfactory receptor, family 4, subfamily C, member 6 |
| rs10897182 | 11 | 55,264,743 | OR4C6 | 55,189,216 | olfactory receptor, family 4, subfamily C, member 6 |
| rs9943519 | 11 | 55,235,453 | OR5D13 | 55,297,490 | olfactory receptor, family 5, subfamily D, member 13 |
| rs2127230 | 11 | 55,253,465 | OR5D13 | 55,297,490 | olfactory receptor, family 5, subfamily D, member 13 |
| rs10897182 | 11 | 55,264,743 | OR5D13 | 55,297,490 | olfactory receptor, family 5, subfamily D, member 13 |
| rs9943519 | 11 | 55,235,453 | OR5D14 | 55,319,608 | olfactory receptor, family 5, subfamily D, member 14 |
| rs2127230 | 11 | 55,253,465 | OR5D14 | 55,319,608 | olfactory receptor, family 5, subfamily D, member 14 |
| rs10897182 | 11 | 55,264,743 | OR5D14 | 55,319,608 | olfactory receptor, family 5, subfamily D, member 14 |
| rs11059100 | 12 | 121,649,582 | GPR109A | 121,710,721 | G protein-coupled receptor 109A |
| rs11059257 | 12 | 121,666,226 | GPR109A | 121,710,721 | G protein-coupled receptor 109A |
| rs11059258 | 12 | 121,666,567 | GPR109A | 121,710,721 | G protein-coupled receptor 109A |
| rs11059100 | 12 | 121,649,582 | GPR109B | 121,724,184 | G protein-coupled receptor 109B |
| rs11059257 | 12 | 121,666,226 | GPR109B | 121,724,184 | G protein-coupled receptor 109B |
| rs11059258 | 12 | 121,666,567 | GPR109B | 121,724,184 | G protein-coupled receptor 109B |
| rs11059100 | 12 | 121,649,582 | GPR81 | 121,735,611 | G protein-coupled receptor 81 |
| rs11059257 | 12 | 121,666,226 | GPR81 | 121,735,611 | G protein-coupled receptor 81 |
| rs11059258 | 12 | 121,666,567 | GPR81 | 121,735,611 | G protein-coupled receptor 81 |
| rs11628803 | 14 | 92,140,517 | RIN3 | 92,049,878 | Ras and Rab interactor 3 |
| rs2146498 | 14 | 92,142,022 | RIN3 | 92,049,878 | Ras and Rab interactor 3 |
| rs4904964 | 14 | 92,169,620 | RIN3 | 92,049,878 | Ras and Rab interactor 3 |
| rs7168345 | 15 | 83,814,605 | AKAP13 | 83,724,875 | A kinase (PRKA) anchor protein 13 |
| rs4514633 | 15 | 83,842,021 | AKAP13 | 83,724,875 | A kinase (PRKA) anchor protein 13 |
| rs4842888 | 15 | 83,864,137 | AKAP13 | 83,724,875 | A kinase (PRKA) anchor protein 13 |
| rs3743321 | 15 | 83,865,972 | AKAP13 | 83,724,875 | A kinase (PRKA) anchor protein 13 |
| rs6496055 | 15 | 83,871,692 | AKAP13 | 83,724,875 | A kinase (PRKA) anchor protein 13 |
| rs12917871 | 16 | 9,714,028 | GRIN2A | 9,762,923 | glutamate receptor, ionotropic, N-methyl D-aspartate 2A |
| rs11074416 | 16 | 9,715,492 | GRIN2A | 9,762,923 | glutamate receptor, ionotropic, N-methyl D-aspartate 2A |
| rs8062216 | 16 | 9,715,998 | GRIN2A | 9,762,923 | glutamate receptor, ionotropic, N-methyl D-aspartate 2A |
| rs6497420 | 16 | 9,716,455 | GRIN2A | 9,762,923 | glutamate receptor, ionotropic, N-methyl D-aspartate 2A |
| rs6497421 | 16 | 9,716,477 | GRIN2A | 9,762,923 | glutamate receptor, ionotropic, N-methyl D-aspartate 2A |
| rs17749793 | 16 | 9,740,694 | GRIN2A | 9,762,923 | glutamate receptor, ionotropic, N-methyl D-aspartate 2A |
| rs8045712 | 16 | 9,758,623 | GRIN2A | 9,762,923 | glutamate receptor, ionotropic, N-methyl D-aspartate 2A |
| rs9911630 | 17 | 38,441,868 | ARHN | 38,430,891 | ras homolog gene family, member N |
| rs8176296 | 17 | 38,457,117 | ARHN | 38,430,891 | ras homolog gene family, member N |
| rs4793197 | 17 | 38,485,428 | ARHN | 38,430,891 | ras homolog gene family, member N |
| rs799916 | 17 | 38,496,716 | ARHN | 38,430,891 | ras homolog gene family, member N |
| rs8176126 | 17 | 38,512,575 | ARHN | 38,430,891 | ras homolog gene family, member N |
| rs4520874 | 17 | 38,771,048 | ARF4L | 38,831,885 | ADP-ribosylation factor 4-like |
| rs4793229 | 17 | 38,773,860 | ARF4L | 38,831,885 | ADP-ribosylation factor 4-like |
| rs9646413 | 17 | 38,781,683 | ARF4L | 38,831,885 | ADP-ribosylation factor 4-like |
| rs4792990 | 17 | 38,817,755 | ARF4L | 38,831,885 | ADP-ribosylation factor 4-like |
| rs11079856 | 17 | 44,545,771 | ABI3 | 44,642,588 | ABI gene family, member 3 |
| rs1554551 | 17 | 44,548,140 | ABI3 | 44,642,588 | ABI gene family, member 3 |
| rs17636326 | 17 | 44,572,905 | ABI3 | 44,642,588 | ABI gene family, member 3 |
| rs6505850 | 18 | 13,839,297 | MC5R | 13,815,765 | melanocortin 5 receptor |
| rs948324 | 18 | 13,842,419 | MC5R | 13,815,765 | melanocortin 5 receptor |
| rs1941092 | 18 | 13,861,956 | MC5R | 13,815,765 | melanocortin 5 receptor |
| rs6505850 | 18 | 13,839,297 | MC2R | 13,874,624 | melanocortin 2 receptor |
| rs948324 | 18 | 13,842,419 | MC2R | 13,874,624 | melanocortin 2 receptor |
| rs1941092 | 18 | 13,861,956 | MC2R | 13,874,624 | melanocortin 2 receptor |
| rs1029224 | 21 | 29,932,927 | GRIK1 | 29,831,127 | glutamate receptor, ionotropic, kainate 1 |
| rs2249140* | 21 | 29,939,211 | GRIK1 | 29,831,127 | glutamate receptor, ionotropic, kainate 1 |
| rs11088125* | 21 | 29,945,889 | GRIK1 | 29,831,127 | glutamate receptor, ionotropic, kainate 1 |
| rs363515 | 21 | 29,964,388 | GRIK1 | 29,831,127 | glutamate receptor, ionotropic, kainate 1 |
|  |  |  |  |  |  |
| ***Channel( n = 5)*** | | | | | |
| rs11264275 | 1 | 151,638,343 | KCNN3 | 151,492,990 | potassium intermediate/small conductance calcium-activated channel, subfamily N, member 3 |
| rs10908448 | 1 | 151,695,617 | KCNN3 | 151,492,990 | potassium intermediate/small conductance calcium-activated channel, subfamily N, member 3 |
| rs4845690 | 1 | 151,699,890 | KCNN3 | 151,492,990 | potassium intermediate/small conductance calcium-activated channel, subfamily N, member 3 |
| rs3767511 | 1 | 197,783,856 | CACNA1S | 197,740,299 | calcium channel, voltage-dependent, L type, alpha 1S subunit |
| rs8158 | 1 | 197,844,638 | CACNA1S | 197,740,299 | calcium channel, voltage-dependent, L type, alpha 1S subunit |
| rs6664337 | 1 | 197,848,411 | CACNA1S | 197,740,299 | calcium channel, voltage-dependent, L type, alpha 1S subunit |
| rs12753507 | 1 | 211,768,747 | KCNK2 | 211,645,031 | potassium channel, subfamily K, member 2 |
| rs946489 | 1 | 211,804,015 | KCNK2 | 211,645,031 | potassium channel, subfamily K, member 2 |
| rs10864169 | 1 | 211,805,836 | KCNK2 | 211,645,031 | potassium channel, subfamily K, member 2 |
| rs4655397 | 1 | 211,805,948 | KCNK2 | 211,645,031 | potassium channel, subfamily K, member 2 |
| rs1526595 | 3 | 54,903,536 | CACNA2D3 | 54,132,528 | calcium channel, voltage-dependent, alpha 2/delta 3 subunit |
| rs9808962 | 3 | 54,916,481 | CACNA2D3 | 54,132,528 | calcium channel, voltage-dependent, alpha 2/delta 3 subunit |
| rs1918070 | 3 | 54,916,618 | CACNA2D3 | 54,132,528 | calcium channel, voltage-dependent, alpha 2/delta 3 subunit |
| rs2048805 | 3 | 54,946,579 | CACNA2D3 | 54,132,528 | calcium channel, voltage-dependent, alpha 2/delta 3 subunit |
| rs17054602 | 3 | 54,959,264 | CACNA2D3 | 54,132,528 | calcium channel, voltage-dependent, alpha 2/delta 3 subunit |
| rs3821659 | 3 | 54,962,963 | CACNA2D3 | 54,132,528 | calcium channel, voltage-dependent, alpha 2/delta 3 subunit |
| rs12052059 | 19 | 13,216,068 | CACNA1A | 13,179,115 | calcium channel, voltage-dependent, P/Q type, alpha 1A subunit |
| rs12611029 | 19 | 13,216,633 | CACNA1A | 13,179,115 | calcium channel, voltage-dependent, P/Q type, alpha 1A subunit |
| rs2302080 | 19 | 13,217,380 | CACNA1A | 13,179,115 | calcium channel, voltage-dependent, P/Q type, alpha 1A subunit |
|  |  |  |  |  |  |
| ***Transcriptional regulator (n = 27)*** | | | | | |
| rs11264275 | 1 | 151,638,343 | PBXIP1 | 151,729,629 | pre-B-cell leukemia transcription factor interacting protein 1 |
| rs10908448 | 1 | 151,695,617 | PBXIP1 | 151,729,629 | pre-B-cell leukemia transcription factor interacting protein 1 |
| rs4845690 | 1 | 151,699,890 | PBXIP1 | 151,729,629 | pre-B-cell leukemia transcription factor interacting protein 1 |
| rs6427042 | 1 | 163,595,214 | STAF42 | 163,557,409 | SPT3-associated factor 42 |
| rs11579987 | 1 | 163,621,022 | STAF42 | 163,557,409 | SPT3-associated factor 42 |
| A-2022484 | 1 | 163,671,399 | STAF42 | 163,557,409 | SPT3-associated factor 42 |
| rs6427042 | 1 | 163,595,214 | C1orf32 | 163,619,654 | chromosome 1 open reading frame 32 LISCH7 homolog |
| rs11579987 | 1 | 163,621,022 | C1orf32 | 163,619,654 | chromosome 1 open reading frame 32 LISCH7 homolog |
| A-2022484 | 1 | 163,671,399 | C1orf32 | 163,619,654 | chromosome 1 open reading frame 32 LISCH7 homolog |
| rs7545911 | 1 | 163,679,339 | C1orf32 | 163,619,654 | chromosome 1 open reading frame 32 LISCH7 homolog |
| rs6682062 | 1 | 163,681,637 | C1orf32 | 163,619,654 | chromosome 1 open reading frame 32 LISCH7 homolog |
| rs6688613 | 1 | 163,683,527 | C1orf32 | 163,619,654 | chromosome 1 open reading frame 32 LISCH7 homolog |
| rs1327866 | 1 | 163,684,568 | C1orf32 | 163,619,654 | chromosome 1 open reading frame 32 LISCH7 homolog |
| rs8158 | 1 | 197,844,638 | DKFZp434B1231 | 197,913,427 | eEF1A2 binding protein |
| rs6664337 | 1 | 197,848,411 | DKFZp434B1231 | 197,913,427 | eEF1A2 binding protein |
| rs12466 | 1 | 197,929,465 | DKFZp434B1231 | 197,913,427 | eEF1A2 binding protein |
| rs2268156* | 1 | 197,998,731 | DKFZp434B1231 | 197,913,427 | eEF1A2 binding protein |
| rs2200243 | 3 | 25,377,048 | RARB | 25,444,758 | retinoic acid receptor, beta |
| rs17523645 | 3 | 25,398,435 | RARB | 25,444,758 | retinoic acid receptor, beta |
| rs4681024 | 3 | 25,450,886 | RARB | 25,444,758 | retinoic acid receptor, beta |
| rs7630905 | 3 | 71,555,237 | FOXP1 | 71,088,616 | forkhead box P1 |
| rs17656368 | 3 | 71,556,906 | FOXP1 | 71,088,616 | forkhead box P1 |
| rs9878602 | 3 | 71,618,028 | FOXP1 | 71,088,616 | forkhead box P1 |
| rs830603 | 3 | 71,717,876 | FOXP1 | 71,088,616 | forkhead box P1 |
| rs3860110 | 5 | 134,896,238 | NEUROG1 | 134,897,873 | neurogenin 1 |
| rs2344485 | 5 | 134,896,540 | NEUROG1 | 134,897,873 | neurogenin 1 |
| rs11739936 | 5 | 134,941,470 | NEUROG1 | 134,897,873 | neurogenin 1 |
| rs17168737 | 5 | 134,971,400 | NEUROG1 | 134,897,873 | neurogenin 1 |
| rs9366344 | 6 | 20,435,828 | E2F3 | 20,510,377 | E2F transcription factor 3 |
| rs9358331 | 6 | 20,452,975 | E2F3 | 20,510,377 | E2F transcription factor 3 |
| rs9356730 | 6 | 20,453,095 | E2F3 | 20,510,377 | E2F transcription factor 3 |
| rs583501 | 6 | 153,288,348 | FBXO5 | 153,383,772 | F-box protein 5 |
| rs681345 | 6 | 153,289,601 | FBXO5 | 153,383,772 | F-box protein 5 |
| rs656610 | 6 | 153,310,375 | FBXO5 | 153,383,772 | F-box protein 5 |
| rs633399 | 6 | 153,312,388 | FBXO5 | 153,383,772 | F-box protein 5 |
| rs17152302 | 8 | 10,657,436 | SOX7 | 10,618,689 | SRY (sex determining region Y)-box 7 |
| rs10113332 | 8 | 10,691,571 | SOX7 | 10,618,689 | SRY (sex determining region Y)-box 7 |
| rs12681861 | 8 | 10,704,353 | SOX7 | 10,618,689 | SRY (sex determining region Y)-box 7 |
| rs559433 | 9 | 4,011,386 | GLIS3 | 3,817,677 | GLIS family zinc finger 3 |
| rs579500 | 9 | 4,016,952 | GLIS3 | 3,817,677 | GLIS family zinc finger 3 |
| rs7028492 | 9 | 4,070,755 | GLIS3 | 3,817,677 | GLIS family zinc finger 3 |
| rs11791374 | 9 | 93,324,842 | PHF2 | 93,418,585 | PHD finger protein 2 |
| rs10821147 | 9 | 93,350,922 | PHF2 | 93,418,585 | PHD finger protein 2 |
| rs4744250 | 9 | 93,351,307 | PHF2 | 93,418,585 | PHD finger protein 2 |
| rs10761238 | 9 | 93,430,772 | PHF2 | 93,418,585 | PHD finger protein 2 |
| rs9409476 | 9 | 93,447,375 | PHF2 | 93,418,585 | PHD finger protein 2 |
| rs4978998 | 9 | 111,298,502 | ZNF483 | 111,366,994 | zinc finger protein 483 |
| rs4448355 | 9 | 111,362,600 | ZNF483 | 111,366,994 | zinc finger protein 483 |
| rs10817185 | 9 | 111,380,600 | ZNF483 | 111,366,994 | zinc finger protein 483 |
| rs9423765 | 10 | 7,152,082 | SFMBT2 | 7,244,255 | Scm-like with four mbt domains 2 |
| rs12771874 | 10 | 7,157,713 | SFMBT2 | 7,244,255 | Scm-like with four mbt domains 2 |
| rs7906988* | 10 | 7,194,153 | SFMBT2 | 7,244,255 | Scm-like with four mbt domains 2 |
| rs629119 | 11 | 93,890,094 | FGIF | 93,866,801 | fetal globin-inducing factor |
| rs629156 | 11 | 93,890,123 | FGIF | 93,866,801 | fetal globin-inducing factor |
| rs16924603 | 11 | 93,894,907 | FGIF | 93,866,801 | fetal globin-inducing factor |
| rs11020812 | 11 | 93,906,765 | FGIF | 93,866,801 | fetal globin-inducing factor |
| rs11020836 | 11 | 93,948,540 | FGIF | 93,866,801 | fetal globin-inducing factor |
| rs629119 | 11 | 93,890,094 | PIWIL4 | 93,940,182 | piwi-like 4 |
| rs629156 | 11 | 93,890,123 | PIWIL4 | 93,940,182 | piwi-like 4 |
| rs16924603 | 11 | 93,894,907 | PIWIL4 | 93,940,182 | piwi-like 4 |
| rs11020812 | 11 | 93,906,765 | PIWIL4 | 93,940,182 | piwi-like 4 |
| rs11020836 | 11 | 93,948,540 | PIWIL4 | 93,940,182 | piwi-like 4 |
| rs10831258 | 11 | 94,026,371 | PIWIL4 | 93,940,182 | piwi-like 4 |
| rs10765698* | 11 | 94,082,118 | PIWIL4 | 93,940,182 | piwi-like 4 |
| rs2285330 | 13 | 31,619,615 | FRY | 31,503,437 | furry homolog hypothetical protein CG003 |
| rs2428249 | 13 | 31,674,603 | FRY | 31,503,437 | furry homolog hypothetical protein CG003 |
| rs798987 | 13 | 31,685,876 | FRY | 31,503,437 | furry homolog hypothetical protein CG003 |
| rs10498318 | 14 | 33,249,118 | NPAS3 | 32,478,200 | neuronal PAS domain protein 3 |
| rs8017055 | 14 | 33,312,734 | NPAS3 | 32,478,200 | neuronal PAS domain protein 3 |
| rs12895061 | 14 | 33,316,845 | NPAS3 | 32,478,200 | neuronal PAS domain protein 3 |
| rs12435979 | 14 | 56,307,190 | OTX2 | 56,337,180 | orthodenticle homolog 2 |
| rs12436671 | 14 | 56,307,786 | OTX2 | 56,337,180 | orthodenticle homolog 2 |
| rs198228* | 14 | 56,312,173 | OTX2 | 56,337,180 | orthodenticle homolog 2 |
| rs2104718* | 14 | 56,395,127 | OTX2 | 56,337,180 | orthodenticle homolog 2 |
| rs17638800 | 16 | 27,089,322 | JMJD5 | 27,122,317 | JMJD5 jumonji domain containing 5 FLJ13798 |
| rs8045153 | 16 | 27,089,979 | JMJD5 | 27,122,317 | JMJD5 jumonji domain containing 5 FLJ13798 |
| rs4787413 | 16 | 27,110,532 | JMJD5 | 27,122,317 | JMJD5 jumonji domain containing 5 FLJ13798 |
| rs9911630 | 17 | 38,441,868 | IFI35 | 38,412,351 | interferon-induced protein 35 |
| rs8176296 | 17 | 38,457,117 | IFI35 | 38,412,351 | interferon-induced protein 35 |
| rs4793197 | 17 | 38,485,428 | IFI35 | 38,412,351 | interferon-induced protein 35 |
| rs799916 | 17 | 38,496,716 | IFI35 | 38,412,351 | interferon-induced protein 35 |
| rs8176126 | 17 | 38,512,575 | IFI35 | 38,412,351 | interferon-induced protein 35 |
| rs9911630 | 17 | 38,441,868 | BRCA1 | 38,449,844 | breast cancer 1, early onset |
| rs8176296 | 17 | 38,457,117 | BRCA1 | 38,449,844 | breast cancer 1, early onset |
| rs4793197 | 17 | 38,485,428 | BRCA1 | 38,449,844 | breast cancer 1, early onset |
| rs799916 | 17 | 38,496,716 | BRCA1 | 38,449,844 | breast cancer 1, early onset |
| rs8176126 | 17 | 38,512,575 | BRCA1 | 38,449,844 | breast cancer 1, early onset |
| rs2271573 | 17 | 38,581,147 | BRCA1 | 38,449,844 | breast cancer 1, early onset |
| rs16941927 | 18 | 21,755,876 | SS18 | 21,850,576 | synovial sarcoma translocation, chromosome 18 |
| rs2879282 | 18 | 21,776,530 | SS18 | 21,850,576 | synovial sarcoma translocation, chromosome 18 |
| rs16942056 | 18 | 21,837,313 | SS18 | 21,850,576 | synovial sarcoma translocation, chromosome 18 |
| rs12052059 | 19 | 13,216,068 | IER2 | 13,124,900 | immediate early response 2 |
| rs12611029 | 19 | 13,216,633 | IER2 | 13,124,900 | immediate early response 2 |
| rs2302080 | 19 | 13,217,380 | IER2 | 13,124,900 | immediate early response 2 |
| rs17123507 | 20 | 30,701,745 | COMMD7 | 30,754,166 | COMM domain containing 7 |
| rs4911252* | 20 | 30,793,776 | COMMD7 | 30,754,166 | COMM domain containing 7 |
| rs2424895* | 20 | 30,799,639 | COMMD7 | 30,754,166 | COMM domain containing 7 |
| rs959996 | 20 | 49,442,816 | NFATC2 | 49,441,312 | nuclear factor of activated T-cells, cytoplasmic, calcineurin-dependent 2 |
| rs2426299 | 20 | 49,451,402 | NFATC2 | 49,441,312 | nuclear factor of activated T-cells, cytoplasmic, calcineurin-dependent 2 |
| rs6067758 | 20 | 49,457,455 | NFATC2 | 49,441,312 | nuclear factor of activated T-cells, cytoplasmic, calcineurin-dependent 2 |
| rs1048546 | 21 | 29,166,748 | ZNF294 | 29,222,337 | zinc finger protein 294 |
| rs2254638 | 21 | 29,178,154 | ZNF294 | 29,222,337 | zinc finger protein 294 |
| rs2248501 | 21 | 29,198,282 | ZNF294 | 29,222,337 | zinc finger protein 294 |
| rs2832137* | 21 | 29,201,887 | ZNF294 | 29,222,337 | zinc finger protein 294 |
| rs2832147 | 21 | 29,266,813 | ZNF294 | 29,222,337 | zinc finger protein 294 |
|  |  |  |  |  |  |
| ***Disease-related (n = 9)*** | | | | | |
| rs1873555 | 2 | 43,691,316 | THADA | 43,369,642 | thyroid adenoma associated |
| rs6731009 | 2 | 43,715,079 | THADA | 43,369,642 | thyroid adenoma associated |
| rs11890152 | 2 | 43,719,019 | THADA | 43,369,642 | thyroid adenoma associated |
| rs17037369 | 3 | 38,103,093 | DLEC1 | 38,055,700 | deleted in lung and esophageal cancer 1 |
| rs6599263* | 3 | 38,119,879 | DLEC1 | 38,055,700 | deleted in lung and esophageal cancer 1 |
| rs6767684 | 3 | 38,160,178 | DLEC1 | 38,055,700 | deleted in lung and esophageal cancer 1 |
| rs933820 | 4 | 37,848,788 | PTTG2 | 37,784,622 | pituitary tumor-transforming 2 |
| rs6820715 | 4 | 37,854,356 | PTTG2 | 37,784,622 | pituitary tumor-transforming 2 |
| rs10018836 | 4 | 37,854,493 | PTTG2 | 37,784,622 | pituitary tumor-transforming 2 |
| rs10018389 | 4 | 37,869,135 | PTTG2 | 37,784,622 | pituitary tumor-transforming 2 |
| rs959694 | 4 | 37,874,761 | PTTG2 | 37,784,622 | pituitary tumor-transforming 2 |
| rs868549 | 6 | 125,068,862 | TCBA1 | 124,166,988 | T-cell lymphoma breakpoint associated target 1 |
| rs2875776 | 6 | 125,081,311 | TCBA1 | 124,166,988 | T-cell lymphoma breakpoint associated target 1 |
| rs1415762 | 6 | 125,081,641 | TCBA1 | 124,166,988 | T-cell lymphoma breakpoint associated target 1 |
| rs6455807 | 6 | 162,706,747 | PARK2 | 161,740,082 | Parkinson disease (autosomal recessive, juvenile) 2, parkin |
| rs7740928 | 6 | 162,706,911 | PARK2 | 161,740,082 | Parkinson disease (autosomal recessive, juvenile) 2, parkin |
| rs6930880 | 6 | 162,716,770 | PARK2 | 161,740,082 | Parkinson disease (autosomal recessive, juvenile) 2, parkin |
| rs10945825 | 6 | 162,779,420 | PARK2 | 161,740,082 | Parkinson disease (autosomal recessive, juvenile) 2, parkin |
| rs1557934 | 7 | 44,795,497 | CCM2 | 44,813,092 | cerebral cavernous malformation 2 |
| rs6969223 | 7 | 44,828,454 | CCM2 | 44,813,092 | cerebral cavernous malformation 2 |
| rs1294966 | 7 | 44,847,039 | CCM2 | 44,813,092 | cerebral cavernous malformation 2 |
| rs3735486 | 7 | 44,878,357 | CCM2 | 44,813,092 | cerebral cavernous malformation 2 |
| rs3757572 | 7 | 44,919,896 | CCM2 | 44,813,092 | cerebral cavernous malformation 2 |
| rs10224439* | 7 | 44,954,161 | CCM2 | 44,813,092 | cerebral cavernous malformation 2 |
| rs2190454 | 11 | 17,490,211 | USH1C | 17,472,018 | Usher syndrome 1C (autosomal recessive, severe) |
| rs2240486 | 11 | 17,495,983 | USH1C | 17,472,018 | Usher syndrome 1C (autosomal recessive, severe) |
| rs2108333 | 11 | 17,506,714 | USH1C | 17,472,018 | Usher syndrome 1C (autosomal recessive, severe) |
| rs201905 | 11 | 32,595,768 | PCID1 | 32,561,963 | PCI domain containing 1 (herpesvirus entry mediator) dendritic cell protein |
| rs11031940 | 11 | 32,676,726 | PCID1 | 32,561,963 | PCI domain containing 1 (herpesvirus entry mediator) dendritic cell protein |
| rs1486582 | 11 | 32,680,449 | PCID1 | 32,561,963 | PCI domain containing 1 (herpesvirus entry mediator) dendritic cell protein |
| rs12592307 | 15 | 25,763,768 | OCA2 | 25,673,628 | oculocutaneous albinism II (pink-eye dilution homolog) |
| rs768547 | 15 | 25,774,538 | OCA2 | 25,673,628 | oculocutaneous albinism II (pink-eye dilution homolog) |
| rs16950482 | 15 | 25,791,843 | OCA2 | 25,673,628 | oculocutaneous albinism II (pink-eye dilution homolog) |
|  |  |  |  |  |  |
| ***Structural proteins (n = 12)*** | | | | | |
| rs12495520 | 3 | 47,782,763 | MAP4 | 47,867,190 | microtubule-associated protein 4 |
| rs3772399 | 3 | 47,842,731 | MAP4 | 47,867,190 | microtubule-associated protein 4 |
| rs319694 | 3 | 47,859,957 | MAP4 | 47,867,190 | microtubule-associated protein 4 |
| rs13103430 | 4 | 17,243,624 | KIAA1276 | 17,310,424 | KIAA1276 ABC_SMC2 Plectin homolog (intermediate filament binding) |
| rs3733576 | 4 | 17,262,972 | KIAA1276 | 17,310,424 | KIAA1276 ABC_SMC2 Plectin homolog (intermediate filament binding) |
| rs6449314 | 4 | 17,264,706 | KIAA1276 | 17,310,424 | KIAA1276 ABC_SMC2 Plectin homolog (intermediate filament binding) |
| rs4698629 | 4 | 17,285,153 | KIAA1276 | 17,310,424 | KIAA1276 ABC_SMC2 Plectin homolog (intermediate filament binding) |
| rs4698634* | 4 | 17,306,461 | KIAA1276 | 17,310,424 | KIAA1276 ABC_SMC2 Plectin homolog (intermediate filament binding) |
| rs9342783 | 6 | 70,909,214 | COL19A1 | 70,633,169 | collagen, type XIX, alpha 1 |
| rs3806051 | 6 | 70,943,464 | COL19A1 | 70,633,169 | collagen, type XIX, alpha 1 |
| rs3793064 | 6 | 70,945,597 | COL19A1 | 70,633,169 | collagen, type XIX, alpha 1 |
| rs9342783 | 6 | 70,909,214 | COL9A1 | 70,982,948 | collagen, type IX, alpha 1 |
| rs3806051 | 6 | 70,943,464 | COL9A1 | 70,982,948 | collagen, type IX, alpha 1 |
| rs3793064 | 6 | 70,945,597 | COL9A1 | 70,982,948 | collagen, type IX, alpha 1 |
| rs1557934 | 7 | 44,795,497 | MYO1G | 44,775,450 | myosin IG |
| rs6969223 | 7 | 44,828,454 | MYO1G | 44,775,450 | myosin IG |
| rs1294966 | 7 | 44,847,039 | MYO1G | 44,775,450 | myosin IG |
| rs3735486 | 7 | 44,878,357 | MYO1G | 44,775,450 | myosin IG |
| rs2912302 | 8 | 75,221,845 | JPH1 | 75,311,997 | junctophilin 1 |
| rs1875064 | 8 | 75,238,861 | JPH1 | 75,311,997 | junctophilin 1 |
| rs2977056 | 8 | 75,247,824 | JPH1 | 75,311,997 | junctophilin 1 |
| rs2936680 | 8 | 75,251,256 | JPH1 | 75,311,997 | junctophilin 1 |
| rs10957702 | 8 | 75,306,307 | JPH1 | 75,311,997 | junctophilin 1 |
| rs2977046 | 8 | 75,342,192 | JPH1 | 75,311,997 | junctophilin 1 |
| rs12251257 | 10 | 91,599,612 | MPHOSPH1 | 91,451,347 | M-phase phosphoprotein 1 |
| rs7079714 | 10 | 91,601,841 | MPHOSPH1 | 91,451,347 | M-phase phosphoprotein 1 |
| rs10881695 | 10 | 91,602,036 | MPHOSPH1 | 91,451,347 | M-phase phosphoprotein 1 |
| rs10509582* | 10 | 91,607,104 | MPHOSPH1 | 91,451,347 | M-phase phosphoprotein 1 |
| rs2190454 | 11 | 17,490,211 | OTOG | 17,556,387 | otogelin |
| rs2240486 | 11 | 17,495,983 | OTOG | 17,556,387 | otogelin |
| rs2108333 | 11 | 17,506,714 | OTOG | 17,556,387 | otogelin |
| rs2512674 | 11 | 82,839,423 | DLG2 | 82,848,327 | discs, large homolog 2, chapsyn-110 |
| rs6592120 | 11 | 82,913,481 | DLG2 | 82,848,327 | discs, large homolog 2, chapsyn-110 |
| rs515618 | 11 | 82,970,299 | DLG2 | 82,848,327 | discs, large homolog 2, chapsyn-110 |
| rs558211 | 11 | 82,974,099 | DLG2 | 82,848,327 | discs, large homolog 2, chapsyn-110 |
| rs948379 | 11 | 120,625,340 | TECTA | 120,478,585 | tectorin alpha |
| rs10892714 | 11 | 120,642,900 | TECTA | 120,478,585 | tectorin alpha |
| rs1583124 | 11 | 120,660,151 | TECTA | 120,478,585 | tectorin alpha |
| rs11058797 | 12 | 121,587,474 | KNTC1 | 121,536,689 | kinetochore associated 1 |
| rs11059100 | 12 | 121,649,582 | KNTC1 | 121,536,689 | kinetochore associated 1 |
| rs11059257 | 12 | 121,666,226 | KNTC1 | 121,536,689 | kinetochore associated 1 |
| rs11059258 | 12 | 121,666,567 | KNTC1 | 121,536,689 | kinetochore associated 1 |
| rs916425 | 22 | 24,712,891 | MYO18B | 24,462,674 | myosin XVIIIB |
| rs916428 | 22 | 24,716,384 | MYO18B | 24,462,674 | myosin XVIIIB |
| rs9613081 | 22 | 24,728,655 | MYO18B | 24,462,674 | myosin XVIIIB |
|  |  |  |  |  |  |
| ***Vesicle (n = 4)*** | | | | | |
| rs16898637 | 8 | 124,710,187 | ANXA13 | 124,762,216 | annexin A13 |
| rs12545556* | 8 | 124,715,203 | ANXA13 | 124,762,216 | annexin A13 |
| rs11777302* | 8 | 124,719,608 | ANXA13 | 124,762,216 | annexin A13 |
| rs13261694 | 8 | 124,722,976 | ANXA13 | 124,762,216 | annexin A13 |
| rs7387544 | 8 | 124,733,973 | ANXA13 | 124,762,216 | annexin A13 |
| rs4374971 | 8 | 124,735,468 | ANXA13 | 124,762,216 | annexin A13 |
| rs6470170 | 8 | 124,736,503 | ANXA13 | 124,762,216 | annexin A13 |
| rs1405493 | 12 | 78,296,432 | SYT1 | 78,113,751 | synaptotagmin I |
| rs7315638 | 12 | 78,309,747 | SYT1 | 78,113,751 | synaptotagmin I |
| rs2251214 | 12 | 78,326,319 | SYT1 | 78,113,751 | synaptotagmin I |
| rs7138252 | 12 | 78,424,787 | SYT1 | 78,113,751 | synaptotagmin I |
| rs580428 | 18 | 10,013,675 | VAPA | 9,903,984 | VAMP (vesicle-associated membrane protein)-associated protein A, 33kDa |
| rs16956115 | 18 | 10,019,871 | VAPA | 9,903,984 | VAMP (vesicle-associated membrane protein)-associated protein A, 33kDa |
| rs9945119 | 18 | 10,025,436 | VAPA | 9,903,984 | VAMP (vesicle-associated membrane protein)-associated protein A, 33kDa |
| rs7245342 | 18 | 10,042,043 | VAPA | 9,903,984 | VAMP (vesicle-associated membrane protein)-associated protein A, 33kDa |
| rs4820483 | 22 | 41,512,678 | PACSIN2 | 41,590,275 | protein kinase C and casein kinase substrate in neurons 2 |
| rs5758965 | 22 | 41,551,097 | PACSIN2 | 41,590,275 | protein kinase C and casein kinase substrate in neurons 2 |
| rs738537 | 22 | 41,602,420 | PACSIN2 | 41,590,275 | protein kinase C and casein kinase substrate in neurons 2 |
| rs12166809 | 22 | 41,611,788 | PACSIN2 | 41,590,275 | protein kinase C and casein kinase substrate in neurons 2 |
| rs4820494 | 22 | 41,630,359 | PACSIN2 | 41,590,275 | protein kinase C and casein kinase substrate in neurons 2 |
| rs1071960 | 22 | 41,674,130 | PACSIN2 | 41,590,275 | protein kinase C and casein kinase substrate in neurons 2 |
|  |  |  |  |  |  |
| ***Transporter (n = 5)*** | | | | | |
| rs2190454 | 11 | 17,490,211 | ABCC8 | 17,371,009 | ATP-binding cassette, sub-family C (CFTR/MRP), member 8 |
| rs2240486 | 11 | 17,495,983 | ABCC8 | 17,371,009 | ATP-binding cassette, sub-family C (CFTR/MRP), member 8 |
| rs2108333 | 11 | 17,506,714 | ABCC8 | 17,371,009 | ATP-binding cassette, sub-family C (CFTR/MRP), member 8 |
| rs3763889 | 11 | 35,263,469 | SLC1A2 | 35,229,329 | solute carrier family 1 (glial high affinity glutamate transporter), member 2 |
| rs11033099 | 11 | 35,345,354 | SLC1A2 | 35,229,329 | solute carrier family 1 (glial high affinity glutamate transporter), member 2 |
| rs7116961 | 11 | 35,348,844 | SLC1A2 | 35,229,329 | solute carrier family 1 (glial high affinity glutamate transporter), member 2 |
| rs943048 | 13 | 24,078,246 | ATP12A | 24,152,695 | ATPase, H+/K+ transporting, nongastric, alpha polypeptide |
| rs1867766 | 13 | 24,159,828 | ATP12A | 24,152,695 | ATPase, H+/K+ transporting, nongastric, alpha polypeptide |
| rs7986361 | 13 | 24,160,942 | ATP12A | 24,152,695 | ATPase, H+/K+ transporting, nongastric, alpha polypeptide |
| rs1048789 | 13 | 25,492,700 | ATP8A2 | 24,941,115 | ATPase, aminophospholipid transporter-like, Class I, type 8A, member 2 |
| rs9512034 | 13 | 25,498,688 | ATP8A2 | 24,941,115 | ATPase, aminophospholipid transporter-like, Class I, type 8A, member 2 |
| rs4770903 | 13 | 25,502,630 | ATP8A2 | 24,941,115 | ATPase, aminophospholipid transporter-like, Class I, type 8A, member 2 |
| rs9911630 | 17 | 38,441,868 | VAT1 | 38,420,148 | vesicle amine transport protein 1 homolog |
| rs8176296 | 17 | 38,457,117 | VAT1 | 38,420,148 | vesicle amine transport protein 1 homolog |
| rs4793197 | 17 | 38,485,428 | VAT1 | 38,420,148 | vesicle amine transport protein 1 homolog |
| rs799916 | 17 | 38,496,716 | VAT1 | 38,420,148 | vesicle amine transport protein 1 homolog |
| rs8176126 | 17 | 38,512,575 | VAT1 | 38,420,148 | vesicle amine transport protein 1 homolog |
|  |  |  |  |  |  |
| ***DNA, RNA, protein processing/turnover (n = 32)*** | | | | | |
| rs6424106 | 1 | 24,643,759 | SRRM1 | 24,714,900 | serine/arginine repetitive matrix 1 |
| rs6687818 | 1 | 24,647,304 | SRRM1 | 24,714,900 | serine/arginine repetitive matrix 1 |
| rs9328948 | 1 | 24,662,762 | SRRM1 | 24,714,900 | serine/arginine repetitive matrix 1 |
| rs6427042 | 1 | 163,595,214 | FLJ14904 | 163,690,177 | FLJ14904 MAEL maelstrom homolog |
| rs11579987 | 1 | 163,621,022 | FLJ14904 | 163,690,177 | FLJ14904 MAEL maelstrom homolog |
| A-2022484 | 1 | 163,671,399 | FLJ14904 | 163,690,177 | FLJ14904 MAEL maelstrom homolog |
| rs7545911 | 1 | 163,679,339 | FLJ14904 | 163,690,177 | FLJ14904 MAEL maelstrom homolog |
| rs6682062 | 1 | 163,681,637 | FLJ14904 | 163,690,177 | FLJ14904 MAEL maelstrom homolog |
| rs6688613 | 1 | 163,683,527 | FLJ14904 | 163,690,177 | FLJ14904 MAEL maelstrom homolog |
| rs1327866 | 1 | 163,684,568 | FLJ14904 | 163,690,177 | FLJ14904 MAEL maelstrom homolog |
| rs12133491 | 1 | 163,788,841 | FLJ14904 | 163,690,177 | FLJ14904 MAEL maelstrom homolog |
| rs10127714 | 1 | 163,798,267 | FLJ14904 | 163,690,177 | FLJ14904 MAEL maelstrom homolog |
| rs7412201 | 1 | 163,821,046 | FLJ14904 | 163,690,177 | FLJ14904 MAEL maelstrom homolog |
| rs486012 | 3 | 9,016,299 | RAD18 | 8,896,561 | RAD18 homolog |
| rs341795 | 3 | 9,021,906 | RAD18 | 8,896,561 | RAD18 homolog |
| rs884235 | 3 | 9,054,481 | RAD18 | 8,896,561 | RAD18 homolog |
| rs7611732 | 3 | 47,755,721 | SMARCC1 | 47,602,391 | SWI/SNF matrix associated, actin dependent regulator of chromatin, subfamily c, member 1 |
| rs12495520 | 3 | 47,782,763 | SMARCC1 | 47,602,391 | SWI/SNF, matrix associated, actin dependent regulator of chromatin, subfamily c, member 1 |
| rs3772399 | 3 | 47,842,731 | SMARCC1 | 47,602,391 | SWI/SNF, matrix associated, actin dependent regulator of chromatin, subfamily c, member 1 |
| rs319694 | 3 | 47,859,957 | SMARCC1 | 47,602,391 | SWI/SNF, matrix associated, actin dependent regulator of chromatin, subfamily c, member 1 |
| rs7611732 | 3 | 47,755,721 | DHX30 | 47,819,655 | DEAH (Asp-Glu-Ala-His) box polypeptide 30 |
| rs12495520 | 3 | 47,782,763 | DHX30 | 47,819,655 | DEAH (Asp-Glu-Ala-His) box polypeptide 30 |
| rs3772399 | 3 | 47,842,731 | DHX30 | 47,819,655 | DEAH (Asp-Glu-Ala-His) box polypeptide 30 |
| rs319694 | 3 | 47,859,957 | DHX30 | 47,819,655 | DEAH (Asp-Glu-Ala-His) box polypeptide 30 |
| rs13103430 | 4 | 17,243,624 | EG1 | 17,292,544 | endothelial-derived gene 1 MED28 mediator of RNA pol II transcription,subunit 28 homolog |
| rs3733576 | 4 | 17,262,972 | EG1 | 17,292,544 | endothelial-derived gene 1 MED28 mediator of RNA pol II transcription,subunit 28 homolog |
| rs6449314 | 4 | 17,264,706 | EG1 | 17,292,544 | endothelial-derived gene 1 MED28 mediator of RNA pol II transcription,subunit 28 homolog |
| rs4698629 | 4 | 17,285,153 | EG1 | 17,292,544 | endothelial-derived gene 1 MED28 mediator of RNA pol II transcription,subunit 28 homolog |
| rs4698634* | 4 | 17,306,461 | EG1 | 17,292,544 | endothelial-derived gene 1 MED28 mediator of RNA pol II transcription,subunit 28 homolog |
| rs4077385 | 4 | 177,603,663 | ASB5 | 177,509,977 | ankyrin repeat and SOCS box-containing 5 |
| rs12331837 | 4 | 177,604,034 | ASB5 | 177,509,977 | ankyrin repeat and SOCS box-containing 5 |
| rs11133144 | 4 | 177,604,868 | ASB5 | 177,509,977 | ankyrin repeat and SOCS box-containing 5 |
| rs4077385 | 4 | 177,603,663 | FLJ22649 | 177,616,264 | SPCS3 signal peptidase complex subunit 3 homolog |
| rs12331837 | 4 | 177,604,034 | FLJ22649 | 177,616,264 | SPCS3 signal peptidase complex subunit 3 homolog |
| rs11133144 | 4 | 177,604,868 | FLJ22649 | 177,616,264 | SPCS3 signal peptidase complex subunit 3 homolog |
| rs1570683 | 6 | 18,447,773 | IBRDC2 | 18,495,686 | IBR domain containing 2 p53-inducible E3 ubiquitin ligase |
| rs1408266 | 6 | 18,530,631 | IBRDC2 | 18,495,686 | IBR domain containing 2 p53-inducible E3 ubiquitin ligase |
| rs568108 | 6 | 18,536,837 | IBRDC2 | 18,495,686 | IBR domain containing 2 p53-inducible E3 ubiquitin ligase |
| rs2484355 | 6 | 86,451,122 | SYNCRIP | 86,380,658 | synaptotagmin binding, cytoplasmic RNA interacting protein |
| rs2095483 | 6 | 86,496,483 | SYNCRIP | 86,380,658 | synaptotagmin binding, cytoplasmic RNA interacting protein |
| rs6454487 | 6 | 86,503,587 | SYNCRIP | 86,380,658 | synaptotagmin binding, cytoplasmic RNA interacting protein |
| rs17152302 | 8 | 10,657,436 | PINX1 | 10,659,883 | PIN2-interacting protein 1 |
| rs10113332 | 8 | 10,691,571 | PINX1 | 10,659,883 | PIN2-interacting protein 1 |
| rs12681861 | 8 | 10,704,353 | PINX1 | 10,659,883 | PIN2-interacting protein 1 |
| rs17128267 | 8 | 19,257,352 | SH2D4A | 19,215,483 | SH2 domain containing 4A |
| rs17128272 | 8 | 19,257,994 | SH2D4A | 19,215,483 | SH2 domain containing 4A |
| rs6586833 | 8 | 19,350,542 | SH2D4A | 19,215,483 | SH2 domain containing 4A |
| A-2049472 | 8 | 67,189,136 | DNAJC5B | 67,096,349 | DnaJ (Hsp40) homolog, subfamily C, member 5 beta |
| rs7831234 | 8 | 67,214,949 | DNAJC5B | 67,096,349 | DnaJ (Hsp40) homolog, subfamily C, member 5 beta |
| rs9694082* | 8 | 67,229,334 | DNAJC5B | 67,096,349 | DnaJ (Hsp40) homolog, subfamily C, member 5 beta |
| rs7822243* | 8 | 67,231,942 | DNAJC5B | 67,096,349 | DnaJ (Hsp40) homolog, subfamily C, member 5 beta |
| rs6999100* | 8 | 67,245,286 | DNAJC5B | 67,096,349 | DnaJ (Hsp40) homolog, subfamily C, member 5 beta |
| A-2049472 | 8 | 67,189,136 | TRIM55 | 67,201,832 | TRIM55 tripartite motif-containing 55 |
| rs7831234 | 8 | 67,214,949 | TRIM55 | 67,201,832 | TRIM55 tripartite motif-containing 55 |
| rs9694082* | 8 | 67,229,334 | TRIM55 | 67,201,832 | TRIM55 tripartite motif-containing 55 |
| rs7822243* | 8 | 67,231,942 | TRIM55 | 67,201,832 | TRIM55 tripartite motif-containing 55 |
| rs6999100* | 8 | 67,245,286 | TRIM55 | 67,201,832 | TRIM55 tripartite motif-containing 55 |
| rs16898637 | 8 | 124,710,187 | FBXO32 | 124,584,540 | F-box protein 32 |
| rs12545556* | 8 | 124,715,203 | FBXO32 | 124,584,540 | F-box protein 32 |
| rs11777302* | 8 | 124,719,608 | FBXO32 | 124,584,540 | F-box protein 32 |
| rs10756757 | 9 | 16,509,926 | BNC2 | 16,408,578 | basonuclin 2 |
| rs10756762 | 9 | 16,553,123 | BNC2 | 16,408,578 | basonuclin 2 |
| rs10810581 | 9 | 16,635,894 | BNC2 | 16,408,578 | basonuclin 2 |
| rs973460 | 9 | 83,896,652 | HNRPK | 83,812,646 | heterogeneous nuclear ribonucleoprotein K |
| rs7033778 | 9 | 83,901,319 | HNRPK | 83,812,646 | heterogeneous nuclear ribonucleoprotein K |
| rs1436932 | 9 | 83,903,397 | HNRPK | 83,812,646 | heterogeneous nuclear ribonucleoprotein K |
| rs973460 | 9 | 83,896,652 | C9orf76 | 83,825,267 | chromosome 9 open reading frame 76 |
| rs7033778 | 9 | 83,901,319 | C9orf76 | 83,825,267 | chromosome 9 open reading frame 76 |
| rs1436932 | 9 | 83,903,397 | C9orf76 | 83,825,267 | chromosome 9 open reading frame 76 |
| rs11791374 | 9 | 93,324,842 | C9orf10OS | 93,288,338 | chromosome 9 open reading frame 10 opposite strand |
| rs10821147 | 9 | 93,350,922 | C9orf10OS | 93,288,338 | chromosome 9 open reading frame 10 opposite strand |
| rs4744250 | 9 | 93,351,307 | C9orf10OS | 93,288,338 | chromosome 9 open reading frame 10 opposite strand |
| rs11791374 | 9 | 93,324,842 | C9orf10 | 93,293,728 | chromosome 9 open reading frame 10 DNA polymerase-transactivated protein 1 |
| rs10821147 | 9 | 93,350,922 | C9orf10 | 93,293,728 | chromosome 9 open reading frame 10 DNA polymerase-transactivated protein 1 |
| rs4744250 | 9 | 93,351,307 | C9orf10 | 93,293,728 | chromosome 9 open reading frame 10 DNA polymerase-transactivated protein 1 |
| rs10761238 | 9 | 93,430,772 | C9orf10 | 93,293,728 | chromosome 9 open reading frame 10 DNA polymerase-transactivated protein 1 |
| rs9409476 | 9 | 93,447,375 | C9orf10 | 93,293,728 | chromosome 9 open reading frame 10 DNA polymerase-transactivated protein 1 |
| rs866624 | 9 | 111,167,251 | KIAA0368 | 111,203,724 | KIAA0368 couples the 26 S proteasome to secretory compartments |
| rs6477821 | 9 | 111,224,584 | KIAA0368 | 111,203,724 | KIAA0368 couples the 26 S proteasome to secretory compartments |
| rs7390341 | 9 | 111,242,812 | KIAA0368 | 111,203,724 | KIAA0368 couples the 26 S proteasome to secretory compartments |
| rs12350676 | 9 | 111,249,685 | KIAA0368 | 111,203,724 | KIAA0368 couples the 26 S proteasome to secretory compartments |
| rs4978998 | 9 | 111,298,502 | KIAA0368 | 111,203,724 | KIAA0368 couples the 26 S proteasome to secretory compartments |
| rs4448355 | 9 | 111,362,600 | KIAA0368 | 111,203,724 | KIAA0368 couples the 26 S proteasome to secretory compartments |
| rs10817185 | 9 | 111,380,600 | KIAA0368 | 111,203,724 | KIAA0368 couples the 26 S proteasome to secretory compartments |
| rs2900132 | 9 | 116,489,192 | TRIM32 | 116,529,163 | tripartite motif-containing 32 E3 ubiquitin ligase |
| rs4837746 | 9 | 116,502,133 | TRIM32 | 116,529,163 | tripartite motif-containing 32 |
| rs10983314 | 9 | 116,554,626 | TRIM32 | 116,529,163 | tripartite motif-containing 32 |
| rs10983319 | 9 | 116,563,020 | TRIM32 | 116,529,163 | tripartite motif-containing 32 |
| rs2296869 | 9 | 132,201,966 | TTF1 | 132,280,493 | transcription termination factor, RNA polymerase I |
| rs2296870* | 9 | 132,202,219 | TTF1 | 132,280,493 | transcription termination factor, RNA polymerase I |
| A-2122450* | 9 | 132,203,239 | TTF1 | 132,280,493 | transcription termination factor, RNA polymerase I |
| rs7868762 | 9 | 132,205,129 | TTF1 | 132,280,493 | transcription termination factor, RNA polymerase I |
| rs11243737 | 9 | 132,258,160 | TTF1 | 132,280,493 | transcription termination factor, RNA polymerase I |
| rs2296869 | 9 | 132,201,966 | SETX | 132,168,837 | senataxin |
| rs2296870* | 9 | 132,202,219 | SETX | 132,168,837 | senataxin |
| A-2122450* | 9 | 132,203,239 | SETX | 132,168,837 | senataxin |
| rs7868762 | 9 | 132,205,129 | SETX | 132,168,837 | senataxin |
| rs11243737 | 9 | 132,258,160 | SETX | 132,168,837 | senataxin |
| rs629119 | 11 | 93,890,094 | MRE11A | 93,790,372 | MRE11 meiotic recombination 11 homolog A |
| rs629156 | 11 | 93,890,123 | MRE11A | 93,790,372 | MRE11 meiotic recombination 11 homolog A |
| rs16924603 | 11 | 93,894,907 | MRE11A | 93,790,372 | MRE11 meiotic recombination 11 homolog A |
| rs11020812 | 11 | 93,906,765 | MRE11A | 93,790,372 | MRE11 meiotic recombination 11 homolog A |
| rs11020836 | 11 | 93,948,540 | MRE11A | 93,790,372 | MRE11 meiotic recombination 11 homolog A |
| rs4772214 | 13 | 98,953,078 | TM9SF2 | 98,951,729 | transmembrane 9 superfamily member 2 |
| rs9585118 | 13 | 98,988,505 | TM9SF2 | 98,951,729 | transmembrane 9 superfamily member 2 |
| rs9585127 | 13 | 99,003,333 | TM9SF2 | 98,951,729 | transmembrane 9 superfamily member 2 |
| rs7323834 | 13 | 110,252,125 | ING1 | 110,163,084 | inhibitor of growth family, member 1 |
| rs7319972 | 13 | 110,254,929 | ING1 | 110,163,084 | inhibitor of growth family, member 1 |
| rs6492314 | 13 | 110,267,411 | ING1 | 110,163,084 | inhibitor of growth family, member 1 |
| rs10483556 | 14 | 49,029,992 | RPS29 | 49,120,053 | ribosomal protein S29 |
| rs8009047 | 14 | 49,038,646 | RPS29 | 49,120,053 | ribosomal protein S29 |
| rs7145941* | 14 | 49,056,851 | RPS29 | 49,120,053 | ribosomal protein S29 |
| rs17638800 | 16 | 27,089,322 | FAM84A | 27,143,827 | NSE1 FAM84A family with sequence similarity 84, member A |
| rs8045153 | 16 | 27,089,979 | FAM84A | 27,143,827 | NSE1 FAM84A family with sequence similarity 84, member A |
| rs4787413 | 16 | 27,110,532 | FAM84A | 27,143,827 | NSE1 FAM84A family with sequence similarity 84, member A |
| rs9911630 | 17 | 38,441,868 | RPL27 | 38,403,972 | ribosomal protein L27 |
| rs8176296 | 17 | 38,457,117 | RPL27 | 38,403,972 | ribosomal protein L27 |
| rs4793197 | 17 | 38,485,428 | RPL27 | 38,403,972 | ribosomal protein L27 |
| rs799916 | 17 | 38,496,716 | RPL27 | 38,403,972 | ribosomal protein L27 |
| rs11079856 | 17 | 44,545,771 | IGF2BP1 | 44,429,802 | IGF-II mRNA-binding protein 1 |
| rs1554551 | 17 | 44,548,140 | IGF2BP1 | 44,429,802 | IGF-II mRNA-binding protein 1 |
| rs17636326 | 17 | 44,572,905 | IGF2BP1 | 44,429,802 | IGF-II mRNA-binding protein 1 |
| rs12052059 | 19 | 13,216,068 | STX10 | 13,116,224 | syntaxin 10 |
| rs12611029 | 19 | 13,216,633 | STX10 | 13,116,224 | syntaxin 10 |
| rs2302080 | 19 | 13,217,380 | STX10 | 13,116,224 | syntaxin 10 |
|  |  |  |  |  |  |
| ***Unknown (n = 34)*** | | | | | |
| rs3767511 | 1 | 197,783,856 | TMEM9 | 197,835,557 | transmembrane protein 9 |
| rs8158 | 1 | 197,844,638 | TMEM9 | 197,835,557 | transmembrane protein 9 |
| rs6664337 | 1 | 197,848,411 | TMEM9 | 197,835,557 | transmembrane protein 9 |
| rs12466 | 1 | 197,929,465 | TMEM9 | 197,835,557 | transmembrane protein 9 |
| rs16853059 | 1 | 200,871,841 | PEPP3 | 200,919,639 | PLEKHA6 pleckstrin homology domain containing, family A member 6 |
| rs6702751 | 1 | 200,943,450 | PEPP3 | 200,919,639 | PLEKHA6 pleckstrin homology domain containing, family A member 6 |
| rs7522769 | 1 | 201,024,341 | PEPP3 | 200,919,639 | PLEKHA6 pleckstrin homology domain containing, family A member 6 |
| rs17557525 | 1 | 220,214,033 | TP53BP2 | 220,274,526 | tumor protein p53 binding protein, 2 |
| rs12088254 | 1 | 220,274,086 | TP53BP2 | 220,274,526 | tumor protein p53 binding protein, 2 |
| rs6678365 | 1 | 220,330,469 | TP53BP2 | 220,274,526 | tumor protein p53 binding protein, 2 |
| rs2817509 | 1 | 242,251,118 | SMYD3 | 242,238,685 | SET and MYND domain containing 3 |
| rs7552469 | 1 | 242,315,625 | SMYD3 | 242,238,685 | SET and MYND domain containing 3 |
| rs10924366 | 1 | 242,353,451 | SMYD3 | 242,238,685 | SET and MYND domain containing 3 |
| rs17597696 | 2 | 33,687,316 | FAM98A | 33,720,380 | DKFZP564F0522 FAM98A family with sequence similarity 98, member A |
| rs17013337 | 2 | 33,694,732 | FAM98A | 33,720,380 | DKFZP564F0522 FAM98A family with sequence similarity 98, member A |
| rs897506 | 2 | 33,698,163 | FAM98A | 33,720,380 | DKFZP564F0522 FAM98A family with sequence similarity 98, member A |
| rs1873555 | 2 | 43,691,316 | KIAA2028 | 43,776,098 | PLEKHH2 pleckstrin homology domain containing, family H (with MyTH4 domain) member 2 |
| rs6731009 | 2 | 43,715,079 | KIAA2028 | 43,776,098 | PLEKHH2 pleckstrin homology domain containing, family H (with MyTH4 domain) member 2 |
| rs11890152 | 2 | 43,719,019 | KIAA2028 | 43,776,098 | PLEKHH2 pleckstrin homology domain containing, family H (with MyTH4 domain) member 2 |
| rs4851884 | 2 | 98,254,310 | MGC26733 | 98,267,972 | hypothetical protein MGC26733 |
| rs1010387 | 2 | 98,281,779 | MGC26733 | 98,267,972 | hypothetical protein MGC26733 |
| rs2122756 | 2 | 98,287,755 | MGC26733 | 98,267,972 | hypothetical protein MGC26733 |
| rs933820 | 4 | 37,848,788 | TBC1D1 | 37,801,502 | TBC1 (tre-2/USP6, BUB2, cdc16) domain family, member 1 |
| rs6820715 | 4 | 37,854,356 | TBC1D1 | 37,801,502 | TBC1 (tre-2/USP6, BUB2, cdc16) domain family, member 1 |
| rs10018836 | 4 | 37,854,493 | TBC1D1 | 37,801,502 | TBC1 (tre-2/USP6, BUB2, cdc16) domain family, member 1 |
| rs10018389 | 4 | 37,869,135 | TBC1D1 | 37,801,502 | TBC1 (tre-2/USP6, BUB2, cdc16) domain family, member 1 |
| rs959694 | 4 | 37,874,761 | TBC1D1 | 37,801,502 | TBC1 (tre-2/USP6, BUB2, cdc16) domain family, member 1 |
| rs13160107 | 5 | 76,753,339 | WDR41 | 76,763,826 | WD repeat domain 41 |
| rs335628 | 5 | 76,765,294 | WDR41 | 76,763,826 | WD repeat domain 41 |
| rs3797642 | 5 | 76,784,682 | WDR41 | 76,763,826 | WD repeat domain 41 |
| rs2160758 | 5 | 93,806,178 | KIAA0825 | 93,514,427 | KIAA0825 protein |
| rs13178274 | 5 | 93,807,278 | KIAA0825 | 93,514,427 | KIAA0825 protein |
| rs9285014 | 5 | 93,821,888 | KIAA0825 | 93,514,427 | KIAA0825 protein |
| rs2160758 | 5 | 93,806,178 | MGC34713 | 93,880,920 | hypothetical protein MGC34713 |
| rs13178274 | 5 | 93,807,278 | MGC34713 | 93,880,920 | hypothetical protein MGC34713 |
| rs9285014 | 5 | 93,821,888 | MGC34713 | 93,880,920 | hypothetical protein MGC34713 |
| rs1018631 | 6 | 40,379,505 | FLJ41649 | 40,420,062 | FLJ41649 protein |
| rs4714329 | 6 | 40,381,435 | FLJ41649 | 40,420,062 | FLJ41649 protein |
| rs715831 | 6 | 40,400,445 | FLJ41649 | 40,420,062 | FLJ41649 protein |
| rs9462616 | 6 | 40,464,159 | FLJ41649 | 40,420,062 | FLJ41649 protein |
| rs1557934 | 7 | 44,795,497 | KIAA0363 | 44,893,276 | KIAA0363 protein |
| rs6969223 | 7 | 44,828,454 | KIAA0363 | 44,893,276 | KIAA0363 protein |
| rs1294966 | 7 | 44,847,039 | KIAA0363 | 44,893,276 | KIAA0363 protein |
| rs3735486 | 7 | 44,878,357 | KIAA0363 | 44,893,276 | KIAA0363 protein |
| rs3757572 | 7 | 44,919,896 | KIAA0363 | 44,893,276 | KIAA0363 protein |
| rs10224439* | 7 | 44,954,161 | KIAA0363 | 44,893,276 | KIAA0363 protein |
| rs2164110 | 7 | 63,135,678 | MGC42415 | 63,133,002 | hypothetical protein MGC42415 |
| rs687547 | 7 | 63,189,079 | MGC42415 | 63,133,002 | hypothetical protein MGC42415 |
| rs10949887 | 7 | 63,196,230 | MGC42415 | 63,133,002 | hypothetical protein MGC42415 |
| rs17768351 | 7 | 63,200,256 | MGC42415 | 63,133,002 | hypothetical protein MGC42415 |
| A-2194416 | 7 | 63,412,986 | FLJ90430 | 63,424,412 | hypothetical protein FLJ90430 |
| rs11764842 | 7 | 63,449,378 | FLJ90430 | 63,424,412 | hypothetical protein FLJ90430 |
| rs11763777 | 7 | 63,519,441 | FLJ90430 | 63,424,412 | hypothetical protein FLJ90430 |
| rs17156320 | 7 | 76,944,761 | FLJ42526 | 76,970,411 | FLJ42526 round spermatid basic protein 1-likerotein |
| rs17807185 | 7 | 76,952,946 | FLJ42526 | 76,970,411 | FLJ42526 round spermatid basic protein 1-likerotein |
| rs12113119 | 7 | 76,959,469 | FLJ42526 | 76,970,411 | FLJ42526 round spermatid basic protein 1-likerotein |
| rs2705295 | 8 | 138,987,482 | FLJ45872 | 138,890,869 | FLJ42526 round spermatid basic protein 1-likerotein |
| rs2705291 | 8 | 138,988,883 | FLJ45872 | 138,890,869 | FLJ42526 round spermatid basic protein 1-likerotein |
| rs2705285 | 8 | 139,012,095 | FLJ45872 | 138,890,869 | FLJ42526 round spermatid basic protein 1-likerotein |
| rs2668129 | 8 | 139,034,394 | FLJ45872 | 138,890,869 | FLJ42526 round spermatid basic protein 1-likerotein |
| A-2270256 | 8 | 139,050,457 | FLJ45872 | 138,890,869 | FLJ42526 round spermatid basic protein 1-likerotein |
| rs7047163 | 9 | 18,772,512 | C9orf94 | 18,816,136 | chromosome 9 open reading frame 94 |
| rs7039188 | 9 | 18,823,014 | C9orf94 | 18,816,136 | chromosome 9 open reading frame 94 |
| rs17233198 | 9 | 18,823,053 | C9orf94 | 18,816,136 | chromosome 9 open reading frame 94 |
| rs10502221 | 11 | 116,332,080 | KIAA0999 | 116,219,330 | KIAA0999 protein |
| rs588918 | 11 | 116,361,852 | KIAA0999 | 116,219,330 | KIAA0999 protein |
| rs7115242 | 11 | 116,413,493 | KIAA0999 | 116,219,330 | KIAA0999 protein |
| rs1351452 | 11 | 116,448,564 | KIAA0999 | 116,219,330 | KIAA0999 protein |
| rs10844055 | 12 | 31,965,995 | FLJ10652 | 32,029,259 | hypothetical protein FLJ10652 |
| rs16919113 | 12 | 32,024,793 | FLJ10652 | 32,029,259 | hypothetical protein FLJ10652 |
| rs10771895 | 12 | 32,030,685 | FLJ10652 | 32,029,259 | hypothetical protein FLJ10652 |
| rs7323834 | 13 | 110,252,125 | ANKRD10 | 110,328,889 | ankyrin repeat domain 10 |
| rs7319972 | 13 | 110,254,929 | ANKRD10 | 110,328,889 | ankyrin repeat domain 10 |
| rs6492314 | 13 | 110,267,411 | ANKRD10 | 110,328,889 | ankyrin repeat domain 10 |
| rs8021033 | 14 | 57,000,298 | C14orf35 | 56,927,024 | chromosome 14 open reading frame 35 |
| rs8021172 | 14 | 57,000,360 | C14orf35 | 56,927,024 | chromosome 14 open reading frame 35 |
| rs1152522* | 14 | 57,018,133 | C14orf35 | 56,927,024 | chromosome 14 open reading frame 35 |
| rs8021033 | 14 | 57,000,298 | C14orf105 | 57,006,348 | chromosome 14 open reading frame 105 |
| rs8021172 | 14 | 57,000,360 | C14orf105 | 57,006,348 | chromosome 14 open reading frame 105 |
| rs1152522 | 14 | 57,018,133 | C14orf105 | 57,006,348 | chromosome 14 open reading frame 105 |
| rs17795775 | 14 | 57,068,620 | C14orf105 | 57,006,348 | chromosome 14 open reading frame 105 |
| rs7184516 | 16 | 26,980,349 | TNT | 26,985,529 | TNT protein |
| rs12926642 | 16 | 26,982,794 | TNT | 26,985,529 | TNT protein |
| rs12927904 | 16 | 26,982,895 | TNT | 26,985,529 | TNT protein |
| rs12444183 | 16 | 80,330,710 | CMIP | 80,036,395 | c-Maf-inducing protein |
| rs6564919 | 16 | 80,374,081 | CMIP | 80,036,395 | c-Maf-inducing protein |
| rs7192802 | 16 | 80,374,523 | CMIP | 80,036,395 | c-Maf-inducing protein |
| rs4580153 | 16 | 80,374,740 | CMIP | 80,036,395 | c-Maf-inducing protein |
| rs9911630 | 17 | 38,441,868 | AARSD1 | 38,356,071 | hypothetical protein MGC2744 |
| rs8176296 | 17 | 38,457,117 | AARSD1 | 38,356,071 | hypothetical protein MGC2744 |
| rs4793197 | 17 | 38,485,428 | AARSD1 | 38,356,071 | hypothetical protein MGC2744 |
| rs9911630 | 17 | 38,441,868 | RUNDC1 | 38,386,108 | RUN domain containing 1 |
| rs8176296 | 17 | 38,457,117 | RUNDC1 | 38,386,108 | RUN domain containing 1 |
| rs4793197 | 17 | 38,485,428 | RUNDC1 | 38,386,108 | RUN domain containing 1 |
| rs799916 | 17 | 38,496,716 | RUNDC1 | 38,386,108 | RUN domain containing 1 |
| rs9911630 | 17 | 38,441,868 | NBR2 | 38,531,153 | neighbor of BRCA1 gene 2 |
| rs8176296 | 17 | 38,457,117 | NBR2 | 38,531,153 | neighbor of BRCA1 gene 2 |
| rs4793197 | 17 | 38,485,428 | NBR2 | 38,531,153 | neighbor of BRCA1 gene 2 |
| rs799916 | 17 | 38,496,716 | NBR2 | 38,531,153 | neighbor of BRCA1 gene 2 |
| rs8176126 | 17 | 38,512,575 | NBR2 | 38,531,153 | neighbor of BRCA1 gene 2 |
| rs2271573 | 17 | 38,581,147 | NBR2 | 38,531,153 | neighbor of BRCA1 gene 2 |
| rs4520874 | 17 | 38,771,048 | TMEM106A | 38,719,420 | MGC20235 transmembrane protein 106A |
| rs4793229 | 17 | 38,773,860 | TMEM106A | 38,719,420 | MGC20235 transmembrane protein 106A |
| rs9646413 | 17 | 38,781,683 | TMEM106A | 38,719,420 | MGC20235 transmembrane protein 106A |
| rs4792990 | 17 | 38,817,755 | TMEM106A | 38,719,420 | MGC20235 transmembrane protein 106A |
| rs16975496 | 18 | 10,847,773 | FLJ34907 | 10,784,068 | hypothetical protein FLJ34907 |
| rs264270 | 18 | 10,915,419 | FLJ34907 | 10,784,068 | hypothetical protein FLJ34907 |
| rs9950334 | 18 | 10,938,670 | FLJ34907 | 10,784,068 | hypothetical protein FLJ34907 |
| rs1389535 | 18 | 29,564,414 | KIAA1713 | 29,412,539 | KIAA1713 protein |
| rs9956918 | 18 | 29,569,876 | KIAA1713 | 29,412,539 | KIAA1713 protein |
| rs6507060* | 18 | 29,576,276 | KIAA1713 | 29,412,539 | KIAA1713 protein |
| rs9947894 | 18 | 29,582,718 | KIAA1713 | 29,412,539 | KIAA1713 protein |
| rs1565784 | 18 | 29,612,489 | KIAA1713 | 29,412,539 | KIAA1713 protein |
| rs12958029 | 18 | 29,642,678 | KIAA1713 | 29,412,539 | KIAA1713 protein |
| rs12457802* | 18 | 29,643,863 | KIAA1713 | 29,412,539 | KIAA1713 protein |
| rs4239383* | 18 | 29,655,064 | KIAA1713 | 29,412,539 | KIAA1713 protein |
| rs2976 | 18 | 29,657,252 | KIAA1713 | 29,412,539 | KIAA1713 protein |
| rs1565784 | 18 | 29,612,489 | NOL4 | 29,686,404 | nucleolar protein 4 |
| rs12958029 | 18 | 29,642,678 | NOL4 | 29,686,404 | nucleolar protein 4 |
| rs12457802* | 18 | 29,643,863 | NOL4 | 29,686,404 | nucleolar protein 4 |
| rs4239383* | 18 | 29,655,064 | NOL4 | 29,686,404 | nucleolar protein 4 |
| rs2976 | 18 | 29,657,252 | NOL4 | 29,686,404 | nucleolar protein 4 |
| rs17123507 | 20 | 30,701,745 | FLJ33706 | 30,684,323 | hypothetical protein FLJ33706 |
| rs4911252* | 20 | 30,793,776 | FLJ33706 | 30,684,323 | hypothetical protein FLJ33706 |
| rs2424895* | 20 | 30,799,639 | FLJ33706 | 30,684,323 | hypothetical protein FLJ33706 |
| rs6020113 | 20 | 35,736,459 | FLJ42133 | 35,738,726 | hypothetical FLJ42133 |
| rs16986890 | 20 | 35,759,937 | FLJ42133 | 35,738,726 | hypothetical FLJ42133 |
| rs6020846* | 20 | 35,839,081 | FLJ42133 | 35,738,726 | hypothetical FLJ42133 |

**Additional file 1:** Successfully-abstinent *vs* unsuccessful abstinence comparisons nominate genes for further studies related to genetic underpinnings of successful quitting. Genes and classes of genes are identified when nominally positive (p < 0.01) SNPs cluster within 0.1Mb of other nominally positive SNPs 1) within the gene’s exons or introns or 2) in 3’ or 5’ flanking sequences that lay within 0.1 Mb of an annotated exon or extensions of the currently-annotated exons as described [22].
